# Use of generalized randomized response model for enhancement of finite population variance: A simulation approach

**DOI:** 10.1371/journal.pone.0315658

**Published:** 2024-12-20

**Authors:** Javid Shabbir, Zabihullah Movaheedi

**Affiliations:** 1 Department of Statistics, University of Wah, Wah Cantt, Islamabad, Pakistan; 2 Faculty of Science, Department of Mathematics, Herat University, Herat, Afghanistan; COMSATS University Islamabad, PAKISTAN

## Abstract

Gupta et al. suggested an improved estimator by using the Diana and Perri model in estimating the finite population variance using the single auxiliary variable. On the same lines, Saleem et al. proposed a new scrambled randomized response model (RRT) based on two auxiliary variables for estimating the finite population variance. Recently Azeem et al. presented a new randomized response model in estimating the finite population variance. It is observed that Bias and MSE of these estimators up to first order of approximation seem to lack sufficient information. In this study, we rectify the bias and *MSE* expressions of the estimators proposed by Gupta et al., Saleem et al. and Azeem et al. Additionally, we suggest a new generalized class of estimators that is more efficient in comparison to the previously considered estimators. A simulation study is conducted to establish the behavior of the estimators. The suggested estimator performs better than the estimators considered by the authors earlier.

## 1. Introduction

Obtaining reliable data, particularly direct and honest response from a respondent is a serious issue which takes a lot of effort and time. Under non-sampling errors, nonresponse may appear due to response bias and nonresponse bias. Consequently, results may be deceptive and misleading, which have a negative impact on decision-making issues. As an alternative, to get around these issues, Warner [1] developed an indigenous model known as randomized response model (RRM). In current situation, it is challenging to gather information on sensitive topics like forced abortion, sexual assault, tax evasion, bribery, corruption, drug addiction, human trafficking, and malpractice etc., because respondents may refuse to give honest answers on such delicate phenomena. So, results may remain erroneous or may be misleading and lead to serious biasedness. To preserve confidentiality and protect respondent’s privacy, another option is to use the RRM.

In our daily lives, we come across a variety of scenarios where unpredictability is involved, such as regular variations in the prices of consumable goods and the significant impact of political instability on stock market crashes. A thorough treatment of variability is essential in such contents. Many authors including Yadav et al. [2], Asghar et al. [3], Sanaullah et al. [4] Niaz et al. [5] and Azeem et al. [6] have investigated the estimation of finite population variance using the scrambled models. Sensitive variable was originally used by Singh et al. [7] to estimate finite population variance under multiplicative scrambled response model. A class of estimators for quantitative sensitivity in quantitative RRM was proposed by Diana and Perri [8]. Ahmad et al. [9] and Gupta et al. [10] suggested some variance estimators using the auxiliary information for sensitive variables. Aloraini et al. [11] introduced several separate and combined variance estimators under stratified sampling for estimation of variance. Azeem et al. [12] suggested a new RRM for mean estimation. Zaman et al. [13] discussed the focus group under RRT to obtain efficient estimators. Some other important references include are: [14–19].

In this paper, we adjusted the bias and mean squared error (MSE) expressions that were previously proposed by Gupta et al. [10], Saleem et al. [20] and Azeem et al. [6] under RRT by utilizing the auxiliary variables. Furthermore, we propose an alternative generalized class of estimators for population variance using generalized RRM.

Diana and Perri [8] suggested a model *Z* = *TY*+*S*, where *T* and *S* are two scrambled variables such that *E*(*S*) = 0 and *E*(*T*) = 1, $E(S^{2})=\sigma_{S}^{2}$ and $E(T^{2})=\sigma_{T}^{2}+1.$ Consider a finite population $\Omega =\{\Omega_{1},\Omega_{2},\ldots \ldots ,\Omega_{N}\},$ where *y*_*i*_, (*x*_1*i*_,*x*_2*i*_ and *z*_*i*_ be the characteristics of the study variable *Y*, the auxiliary variables (*X*_1_,*X*_2_) and *Z* respectively. We draw a sample of size *n* from a population by using simple random sampling with replacement (SRSWR). Let $s_{y}^{2}=\frac{1}{n-1}\sum_{i=1}^{n}{\left(y_{i}-\bar{y}\right)}^{2},$ $s_{x_{1}}^{2}=\frac{1}{n-1}\sum_{i=1}^{n}{\left(x_{1i}-{\bar{x}}_{1}\right)}^{2},$ $s_{x_{2}}^{2}=\frac{1}{n-1}\sum_{i=1}^{n}{\left(x_{2i}-{\bar{x}}_{2}\right)}^{2}$ and $s_{z}^{2}=\frac{1}{n-1}\sum_{i=1}^{n}{\left(z_{i}-\bar{z}\right)}^{2}$ be the sample variances corresponding to population variances $\sigma_{y}^{2}=\frac{1}{N-1}\sum_{i=1}^{N}{\left(y_{i}-\bar{Y}\right)}^{2}$, $\sigma_{x_{1}}^{2}=\frac{1}{N-1}\sum_{i=1}^{N}{\left(x_{1i}-{\bar{X}}_{1}\right)}^{2}$, $\sigma_{x_{2}}^{2}=\frac{1}{N-1}\sum_{i=1}^{N}{\left(x_{2i}-{\bar{X}}_{2}\right)}^{2}$ and $\sigma_{z}^{2}=\frac{1}{N-1}\sum_{i=1}^{N}{\left(z_{i}-\bar{Z}\right)}^{2}$ respectively, where $\bar{y}=\frac{1}{n}\sum_{i=1}^{n}y_{i},$ $\bar{x}=\frac{1}{n}\sum_{i=1}^{n}x_{i},$ $\bar{z}=\frac{1}{n}\sum_{i=1}^{n}z_{i},$ and $\bar{Y}=\frac{1}{N}\sum_{i=1}^{N}y_{i},$ $\bar{X}=\frac{1}{N}\sum_{i=1}^{N}x_{i},$ $\bar{Z}=\frac{1}{N}\sum_{i=1}^{N}z_{i}.$ Let we define the error terms to obtain the bias and *MSE* of estimators. Let $E(\zeta_{z})=\frac{s_{z}^{2}-\sigma_{z}^{2}}{\sigma_{z}^{2}},$ $\zeta_{z}^{*}=\frac{\bar{z}-\bar{Z}}{\bar{Z}},$ $\zeta_{x_{1}}^{2}=\frac{s_{x_{1}}^{2}-\sigma_{x_{1}}^{2}}{\sigma_{x_{1}}^{2}},$ $\zeta_{x_{2}}^{2}=\frac{s_{x_{2}}^{2}-\sigma_{x_{2}}^{2}}{\sigma_{x_{2}}^{2}},$ such that $E(\zeta_{z})=E(\zeta_{z}^{*})=E(\zeta_{x_{1}})=E(\zeta_{x_{2}})=0,$ $E(\zeta_{z}^{2})=\theta \gamma_{400}^{*},$ $E(\zeta_{z}^{*2})=\theta C_{z}^{2},$ $E(\zeta_{x_{1}}^{2})=\theta \gamma_{040}^{*},$ $E(\zeta_{x_{2}}^{2})=\theta \gamma_{004}^{*},$ $E(\zeta_{z}\zeta_{z}^{*})=\theta C_{z}\gamma_{300},$ $E(\zeta_{z}\zeta_{x_{1}})=\theta \gamma_{220}^{*},$ $E(\zeta_{z}\zeta_{x_{2}})=\theta \gamma_{202}^{*},$ $E(\zeta_{x_{1}}\zeta_{x_{2}})=\theta \gamma_{022}^{*},$ $E(\zeta_{x_{1}}\zeta_{z}^{*})=\theta \gamma_{120}C_{z},$ $E(\zeta_{x_{2}}\zeta_{z}^{*})=\theta \gamma_{102}C_{z},$ $\theta =\frac{1}{n},$ $\gamma_{rst}=\frac{\mu_{rst}}{\mu_{200}^{r/2}\mu_{020}^{s/2}\mu_{002}^{s/2}},$ $\mu_{rst}=\frac{1}{N-1}\sum_{i=1}^{N}{\left(z_{i}-\bar{Z}\right)}^{r}{\left(x_{1i}-{\bar{X}}_{1}\right)}^{s}{\left(x_{2i}-{\bar{X}}_{2}\right)}^{t}$ and $\gamma_{rst}^{*}=\gamma_{rst}-1.$

## 2. Existing estimators

We discuss the different estimators suggested by different authors under different models.

### (i) Usual variance estimator under Dian and Perri [8] model

The finite population variance of *Z*, is given by

$$\sigma_{z}^{2}=\sigma_{y}^{2}(\sigma_{T}^{2}+1)+\sigma_{S}^{2}+\sigma_{T}^{2}{\bar{Z}}^{2},$$ (1)

where $\sigma_{S}^{2}$ and $\sigma_{T}^{2}$ are the variances of the scrambled variables *S* and *T* respectively. From (1), we can write $\sigma_{y}^{2}$ as $\sigma_{y(0)}^{2}=\frac{1}{\sigma_{T}^{2}+1}\{\sigma_{z}^{2}-\sigma_{S}^{2}-\sigma_{T}^{2}{\bar{Z}}^{2}\}$.

Replacing $\sigma_{z}^{2}$ and ${\bar{Z}}^{2}$ by their consistent estimates $s_{z}^{2}$ and ${\bar{z}}^{2}$ respectively, Gupta et al. [10] proposed an estimator for population variance, is given by:

$${\hat{\sigma }}_{G(0)}^{2}=\frac{1}{\sigma_{T}^{2}+1}\{s_{z}^{2}-\sigma_{S}^{2}-\sigma_{T}^{2}{\bar{z}}^{2}\}.$$ (2)

The bias and *MSE* of ${\hat{\sigma }}_{G(0)}^{2}$ to first order of approximation, is given by

$$Bias({\hat{\sigma }}_{G(0)}^{2})\approx -\theta \frac{\sigma_{T}^{2}{\bar{Z}}^{2}C_{z}^{2}}{\sigma_{T}^{2}+1},$$ (3)

where $C_{z}^{2}=C_{y}^{2}(\sigma_{T}^{2}+1)+\sigma_{T}^{2}+(\sigma_{S}^{2}/{\bar{Y}}^{2})$ as $\bar{Z}=\bar{Y},$ and

$$MSE({\hat{\sigma }}_{G(0)}^{2})\approx \frac{\theta }{{\left(\sigma_{T}^{2}+1\right)}^{2}}[\sigma_{z}^{4}\gamma_{400}^{*}+4\sigma_{T}^{4}{\bar{Z}}^{4}C_{z}^{2}-4\sigma_{z}^{2}\sigma_{T}^{2}{\bar{Z}}^{2}\gamma_{300}C_{z}].$$ (4)

### (ii) Ratio estimator under Dian and Perri [8] model

Gupta et al. [10] defined the following ratio estimator under RRT, is given by:

$${\hat{\sigma }}_{G(R)}^{2}=[\frac{1}{\sigma_{T}^{2}+1}\{s_{z}^{2}-\sigma_{S}^{2}-\sigma_{T}^{2}{\bar{z}}^{2}\}][\frac{\sigma_{x_{1}}^{2}}{s_{x_{1}}^{2}}].$$ (5)

The bias and *MSE* of ${\hat{\sigma }}_{G(R)}^{2}$ to first order of approximation as reported by Gupta et al. [10] are given by

$$Bias({\hat{\sigma }}_{G(R)}^{2})\approx \frac{\theta }{\left(\sigma_{T}^{2}+1\right)}[2\sigma_{T}^{2}{\bar{Z}}^{2}\gamma_{120}C_{z}-\sigma_{z}^{2}\gamma_{220}^{*}-\sigma_{T}^{2}{\bar{Z}}^{2}C_{z}^{2}]$$ (6)

and

$$MSE({\hat{\sigma }}_{G(R)}^{2})\approx \frac{\theta }{{\left(\sigma_{T}^{2}+1\right)}^{2}}[\sigma_{z}^{4}\gamma_{400}^{*}-2\sigma_{z}^{2}\sigma_{y}^{2}\gamma_{220}^{*}(\sigma_{T}^{2}+1)+\sigma_{y}^{4}\gamma_{040}^{*}{\left(\sigma_{T}^{2}+1\right)}^{2}$$

$$+4C_{z}\{\sigma_{T}^{4}{\bar{Z}}^{4}C_{z}-\sigma_{z}^{2}\sigma_{T}^{2}{\bar{Z}}^{2}\gamma_{300}+\sigma_{T}^{2}\sigma_{y}^{2}{\bar{Z}}^{2}\gamma_{120}(\sigma_{T}^{2}+1)\}].$$ (7)

**Note: It is observed that the bias and *MSE* expressions of ${\hat{\sigma }}_{G(R)}^{2}$ to first order of approximation given in (6) and (7) are incorrect**.

Now, we reconsider the estimator ${\hat{\sigma }}_{G(R)}^{2}$ as observed in (5) by giving a name ${\hat{\sigma }}_{G(R)C}^{2}$ and in terms of errors, we have:

$${\hat{\sigma }}_{G(R)C}^{2}-\sigma_{y(0)}^{2}\approx \frac{1}{\left(\sigma_{T}^{2}+1\right)}[\sigma_{z}^{2}\{\zeta_{z}-\zeta_{x}+\zeta_{x_{1}}^{2}-\zeta_{x_{1}}\zeta_{z})\}-\sigma_{S}^{2}\{-\zeta_{x_{1}}+\zeta_{x_{1}}^{2}\}$$

$$-\sigma_{T}^{2}{\bar{Z}}^{2}\{2\zeta_{z}^{*}-\zeta_{x_{1}}-2\zeta_{x_{1}}\zeta_{z}^{*}+\zeta_{z}^{*2}+\zeta_{x_{1}}^{2}\}].$$ (8)

Using (8), the corrected bias and corrected *MSE* of ${\hat{\sigma }}_{G(R)C}^{2}$, are given by:

$$Bias({\hat{\sigma }}_{G(R)C}^{2})\approx \frac{\theta }{\left(\sigma_{T}^{2}+1\right)}[\sigma_{z}^{2}(\gamma_{040}^{*}-\gamma_{220}^{*})-\sigma_{S}^{2}\gamma_{040}^{*}-\sigma_{T}^{2}{\bar{Z}}^{2}(C_{z}^{2}+\gamma_{040}^{*}-2\gamma_{120}C_{z})]$$ (9)

and

$$MSE({\hat{\sigma }}_{G(R)C}^{2})\approx \frac{\theta }{{\left(\sigma_{T}^{2}+1\right)}^{2}}[\sigma_{z}^{4}(\gamma_{040}^{*}+\gamma_{400}^{*}-2\gamma_{220}^{*})+\sigma_{S}^{4}\gamma_{040}^{*}$$

$$+\sigma_{T}^{4}Z^{4}(\gamma_{040}^{*}+4C_{z}^{2}-4\gamma_{120}C_{z})-2\sigma_{z}^{2}\sigma_{S}^{2}(\gamma_{040}^{*}-\gamma_{220}^{*})$$

$$-2\sigma_{T}^{2}\sigma_{z}^{2}{\bar{Z}}^{2}(\gamma_{040}^{*}-\gamma_{220}^{*}-2\gamma_{120}C_{z}+2\gamma_{300}C_{z})+2\sigma_{T}^{2}\sigma_{S}^{2}{\bar{Z}}^{2}(\gamma_{040}^{*}-2\gamma_{120}C_{z})].$$ (10)

### (iii) Generalized ratio type estimator under Diana and Perri [8] model

Gupta et al. [10] suggested another ratio type estimator for population variance as given by:

$${\hat{\sigma }}_{G(G)}^{2}=[\frac{1}{\sigma_{T}^{2}+1}\{s_{z}^{2}-\sigma_{S}^{2}-\sigma_{T}^{2}{\bar{z}}^{2}\}+(\sigma_{x_{1}}^{2}-s_{x_{1}}^{2})][{\left\{\frac{\alpha \sigma_{x_{1}}^{2}+\beta }{w(\alpha s_{x_{1}}^{2}+\beta )+(1-w)(\alpha \sigma_{x_{1}}^{2}+\beta )}\right\}}^{g}],$$ (11)

where *g*,*α*,*β* and *w* are suitably constants.

The bias and minimum *MSE* of ${\hat{\sigma }}_{G(G)}^{2}$ to first order of approximation as reported by Gupta et al. [10], are given by:

$$Bias({\hat{\sigma }}_{G(G)}^{2})\approx -\frac{\theta }{\left(\sigma_{T}^{2}+1\right)}[\sigma_{T}^{2}{\bar{Z}}^{2}C_{z}^{2}+\varphi \{\sigma_{z}^{2}\gamma_{220}^{*}-2\sigma_{T}^{2}{\bar{Z}}^{2}\gamma_{120}C_{z}-\sigma_{x_{1}}^{2}\gamma_{040}^{*}(\sigma_{T}^{2}+1)\}],$$ (12)

where *φ* = *gwψ*_*i*_ and $\psi_{i}=\frac{\alpha \sigma_{x}^{2}}{\alpha \sigma_{x}^{2}+\beta }.$

and

$$MSE{\left({\hat{\sigma }}_{G(G)}^{2}\right)}_{\min}\approx \frac{\theta }{{\left(\sigma_{T}^{2}+1\right)}^{2}}\left[\{\sigma_{z}^{4}\gamma_{40}^{*}+4\sigma_{T}^{4}{\bar{Z}}^{4}C_{z}^{2}-4\sigma_{z}^{2}\sigma_{T}^{2}{\bar{Z}}^{2}\gamma_{30}C_{z}\right\}$$

$$-\frac{1}{\gamma_{040}^{*}}{\left\{\sigma_{z}^{2}\gamma_{220}^{*}-2\sigma_{T}^{2}{\bar{Z}}^{2}\gamma_{120}C_{z}\right\}}^{2}].$$ (13)

The optimum value of *φ*, is given by

$$\varphi_{opt}=\frac{1}{\sigma_{y}^{2}}(\frac{\sigma_{z}^{2}\gamma_{220}^{*}-2\sigma_{T}^{2}{\bar{Z}}^{2}\gamma_{120}C_{z}}{(\sigma_{T}^{2}+1)\gamma_{040}^{*}}-\sigma_{x_{1}}^{2}).$$

**Note: It is also observed that the bias and *MSE* expressions of ${\hat{\sigma }}_{G(G)}^{2}$ given in (12) and (13) to first order of approximation as reported by Gupta et al. [10] are not correct. The corrected bias and *MSE* expressions of ${\hat{\sigma }}_{G(G)}^{2}$ called it ${\hat{\sigma }}_{G(G)C}^{2}$ to first order of approximation, are given below**.

Solving (11) in terms of errors, we have

$$({\hat{\sigma }}_{G(G)C}^{2})\approx [\frac{1}{\left(\sigma_{T}^{2}+1\right)}\{\sigma_{z}^{2}(1+\zeta_{z})-\sigma_{S}^{2}-\sigma_{T}^{2}{\bar{Z}}^{2}{\left(1+\zeta_{z}^{*}\right)}^{2}\}-\sigma_{x_{1}}^{2}\zeta_{x_{1}}]{\left[1+w\psi_{i}\zeta_{x_{1}}\right]}^{-g}.$$ (14)

To first order of approximation, we have

$$({\hat{\sigma }}_{G(G)C}^{2})-\sigma_{y(0)}^{2}\approx \frac{1}{\left(\sigma_{T}^{2}+1\right)}\left[\sigma_{z}^{2}\{\zeta_{z}-w\psi_{i}g\zeta_{x_{1}}-w\psi_{i}g\zeta_{x}\zeta_{z}+\frac{g(g+1)}{2}w^{2}\psi_{i}^{2}\zeta_{x_{1}}^{2}\right\}$$

$$-\sigma_{S}^{2}\{-w\psi_{i}g\zeta_{x_{1}}+\frac{g(g+1)}{2}w^{2}\psi_{i}^{2}\zeta_{x_{1}}^{2}\}$$

$$-\sigma_{T}^{2}{\bar{Z}}^{2}\{\zeta_{z}^{*2}+2\zeta_{z}^{*}-w\psi_{i}g\zeta_{x_{1}}+\frac{g(g+1)}{2}w^{2}\psi_{i}^{2}\zeta_{x_{1}}^{2}-2w\psi_{i}g\zeta_{x_{1}}\zeta_{z}^{*}\}$$

$$-\sigma_{x_{1}}^{2}(\zeta_{x_{1}}-w\psi_{i}g\zeta_{x_{1}}^{2})(\sigma_{T}^{2}+1)].$$ (15)

From (15), the corrected bias expression of ${\hat{\sigma }}_{G(G)C}^{2}$ to first order of approximation, is given by

$$Bias({\hat{\sigma }}_{G(G)C}^{2})\approx \frac{\theta }{\left(\sigma_{T}^{2}+1\right)}\left[\sigma_{z}^{2}\{-w\psi_{i}g\gamma_{220}^{*}+\frac{g(g+1)}{2}w^{2}\psi_{i}^{2}\gamma_{040}^{*}\right\}$$

$$-\sigma_{S}^{2}\frac{g(g+1)}{2}w^{2}\psi_{i}^{2}\gamma_{040}^{*}-\sigma_{T}^{2}{\bar{Z}}^{2}\{C_{z}^{2}+\frac{g(g+1)}{2}w^{2}\psi_{i}^{2}\gamma_{040}^{*}-2w\psi_{i}g\gamma_{120}C_{z}\}$$

$$+\sigma_{x}^{2}w\psi_{i}g\gamma_{040}^{*}(\sigma_{T}^{2}+1)].$$ (16)

Squaring (15) and then taking expectation, we get the corrected *MSE* of ${\hat{\sigma }}_{G(G)C}^{2}$ to first order of approximation, is given by

$$MSE({\hat{\sigma }}_{G(G)C}^{2})\approx \frac{\theta }{{\left(\sigma_{T}^{2}+1\right)}^{2}}[\delta_{1}+w^{2}\psi_{i}^{2}g^{2}\delta_{2}-2w\psi_{i}g\delta_{3}],$$ (17)

where

$$\delta_{1}=(\sigma_{z}^{4}\gamma_{400}^{*}+4\sigma_{T}^{4}{\bar{Z}}^{4}C_{z}^{2}+\sigma_{x}^{4}{\left(\sigma_{T}^{2}+1\right)}^{2}\gamma_{040}^{*}-4\sigma_{z}^{2}\sigma_{T}^{2}\gamma_{300}C_{z}{\bar{Z}}^{2})$$

$$(-2\sigma_{z}^{2}\sigma_{x_{1}}^{2}(\sigma_{T}^{2}+1)\gamma_{220}^{*}+4\sigma_{T}^{2}\sigma_{x_{1}}^{2}{\bar{Z}}^{2}\gamma_{120}C_{z}(\sigma_{T}^{2}+1)),$$

$$\delta_{2}=\gamma_{040}^{*}(\sigma_{z}^{4}+\sigma_{S}^{4}+\sigma_{T}^{4}{\bar{Z}}^{4}-2\sigma_{z}^{2}\sigma_{S}^{2}-2\sigma_{z}^{2}\sigma_{T}^{2}{\bar{Z}}^{2}+2\sigma_{S}^{2}\sigma_{T}^{2}{\bar{Z}}^{2}),$$

$$\delta_{3}=\gamma_{220}^{*}(\sigma_{z}^{4}-\sigma_{z}^{2}\sigma_{S}^{2}-\sigma_{z}^{2}\sigma_{T}^{2}{\bar{Z}}^{2})+2\gamma_{120}C_{z}(\sigma_{T}^{4}{\bar{Z}}^{4}-{\bar{Z}}^{2}\sigma_{z}^{2}\sigma_{T}^{2}+\sigma_{S}^{2}\sigma_{T}^{2}{\bar{Z}}^{2})$$

$$+\gamma_{040}^{*}(\sigma_{T}^{2}+1)\sigma_{x_{1}}^{2}(-\sigma_{z}^{2}+\sigma_{S}^{2}+\sigma_{T}^{2}{\bar{Z}}^{2}).$$

Differentiate (17) w.r.t (*wψ*_*i*_*g*), we get the optimum value as given by

$${\left(w\psi_{i}g\right)}_{opt}=\frac{\delta_{3}}{\delta_{2}}.$$

Substituting (*wψ*_*i*_*g*)_*opt*_ in (17), we get the minimum *MSE* of ${\hat{\sigma }}_{G(G)C}^{2}$ as given by

$$MSE{\left({\hat{\sigma }}_{G(G)C}^{2}\right)}_{\min}\approx \frac{\theta }{{\left(\sigma_{T}^{2}+1\right)}^{2}}[\delta_{1}-\frac{\delta_{3}^{2}}{\delta_{2}}].$$ (18)

### (iv) Difference type estimator using single auxiliary variable

A difference type estimator using a single auxiliary variable, is given by:

$${\hat{\sigma }}_{D_{1}}^{2}=[\frac{1}{\sigma_{T}^{2}+1}\{s_{z}^{2}-\sigma_{S}^{2}-\sigma_{T}^{2}{\bar{z}}^{2}\}+K(\sigma_{x_{1}}^{2}-s_{x_{1}}^{2})],$$ (19)

where *K* is constant.

Solving (19) in terms of errors, we get

$${\hat{\sigma }}_{D_{1}}^{2}-\sigma_{y(0)}^{2}\approx \frac{1}{\left(\sigma_{T}^{2}+1\right)}[\sigma_{z}^{2}\zeta_{z}-\sigma_{T}^{2}{\bar{Z}}^{2}(2\zeta_{z}^{*}+\zeta_{z}^{*2})-K\sigma_{x_{1}}^{2}(\sigma_{T}^{2}+1)\zeta_{z}].$$ (20)

From (20), the bias of ${\hat{\sigma }}_{D_{1}}^{2}$, is given by

$$Bias({\hat{\sigma }}_{D_{1}}^{2})\approx -\frac{\theta }{\left(\sigma_{T}^{2}+1\right)}\sigma_{T}^{2}{\bar{Z}}^{2}C_{z}^{2}.$$ (21)

Squaring (20) and then taking expectation, we get the *MSE* of ${\hat{\sigma }}_{D_{1}}^{2}$ as given by

$$MSE({\hat{\sigma }}_{D_{1}}^{2})\approx \frac{\theta }{{\left(\sigma_{T}^{2}+1\right)}^{2}}[\pi_{1}-2K(\sigma_{T}^{2}+1)\sigma_{x_{1}}^{2}\pi_{2}+K^{2}\sigma_{x_{1}}^{4}{\left(\sigma_{T}^{2}+1\right)}^{2}\gamma_{040}^{*}],$$ (22)

where $\pi_{1}=\sigma_{z}^{4}\gamma_{400}^{*}+4\sigma_{T}^{4}{\bar{Z}}^{4}C_{z}^{2}-4\sigma_{z}^{2}\sigma_{T}^{2}\gamma_{300}C_{z}{\bar{Z}}^{2}$ and $\pi_{2}=\sigma_{z}^{2}\gamma_{220}^{*}-\sigma_{T}^{2}{\bar{Z}}^{2}\gamma_{120}C_{z}$.

From (22), the optimum value of *K* is $K_{opt}=\frac{\pi_{2}}{\sigma_{x_{1}}^{2}(\sigma_{T}^{2}+1)\gamma_{040}^{*}}$.

Substituting the optimum value of *K* in (22), we get minimum *MSE* of ${\hat{\sigma }}_{D_{1}}^{2}$ as given by

$$MSE{\left({\hat{\sigma }}_{D_{1}}^{2}\right)}_{\min}\approx \frac{\theta }{{\left(\sigma_{T}^{2}+1\right)}^{2}}[\pi_{1}-\frac{\pi_{2}^{2}}{\gamma_{040}^{*}}].$$ (23)

### (v) Difference type estimator using two auxiliary variables

A difference type estimator using two auxiliary variables, is given by:

$${\hat{\sigma }}_{D_{2}}^{2}=[J_{1}\frac{1}{\sigma_{T}^{2}+1}\{s_{z}^{2}-\sigma_{S}^{2}-\sigma_{T}^{2}{\bar{z}}^{2}\}+J_{2}(\sigma_{x_{1}}^{2}-s_{x_{1}}^{2})+J_{3}(\sigma_{x_{2}}^{2}-s_{x_{2}}^{2})],$$ (24)

where *J*_*i*_(*i* = 1,2,3) are constants.

Solving (24) in terms of errors, we get

$${\hat{\sigma }}_{D_{2}}^{2}-\sigma_{y(0)}^{2}\approx \frac{1}{\left(\sigma_{T}^{2}+1\right)}\left[(J_{1}-1)\sigma_{y(0)}^{*2}+J_{1}\{\sigma_{z}^{2}\zeta_{z}-\sigma_{T}^{2}{\bar{Z}}^{2}(2\zeta_{z}^{*}+\zeta_{z}^{*2})\right\}$$

$$-J_{2}\sigma_{x_{1}}^{2}(\sigma_{T}^{2}+1)\zeta_{x_{1}}-J_{3}\sigma_{x_{2}}^{2}(\sigma_{T}^{2}+1)\zeta_{x_{2}}],$$ (25)

where $\sigma_{y(0)}^{*2}=S_{z}^{2}-\sigma_{S}^{2}-\sigma_{T}^{2}{\bar{Z}}^{2}$.

From (25), the bias of ${\hat{\sigma }}_{D_{2}}^{2}$, is given by

$$Bias({\hat{\sigma }}_{D_{2}}^{2})\approx \frac{1}{\left(\sigma_{T}^{2}+1\right)}[(J_{1}-1)\sigma_{y(0)}^{*2}-J_{1}\theta \sigma_{T}^{2}{\bar{Z}}^{2}C_{z}^{2}].$$ (26)

Squaring (25) and then taking expectation, we get the *MSE* of ${\hat{\sigma }}_{D_{2}}^{2}$ as given by

$$MSE({\hat{\sigma }}_{D_{2}}^{2})\approx \frac{1}{{\left(\sigma_{T}^{2}+1\right)}^{2}}[\sigma_{y(0)}^{*4}+J_{1}^{2}U_{1}+J_{2}^{2}U_{2}+J_{3}^{2}U_{3}-2J_{1}U_{4}$$

$$-2J_{1}J_{2}U_{5}-2J_{1}J_{3}U_{6}+2J_{2}J_{3}U_{7}],$$ (27)

where $U_{1}=\sigma_{y(0)}^{*4}+\theta (\sigma_{z}^{4}\gamma_{400}^{*}+4\sigma_{T}^{4}{\bar{Z}}^{4}C_{z}^{2}-4\sigma_{z}^{2}\sigma_{T}^{2}\gamma_{300}C_{z}{\bar{Z}}^{2}-2\sigma_{y(0)}^{*2}\sigma_{T}^{2}{\bar{Z}}^{2}C_{z}^{2}),$ $U_{2}=\theta (\sigma_{x_{1}}^{4}{\left(\sigma_{T}^{2}+1\right)}^{2}\gamma_{040}^{*}),\;U_{3}=\theta (\sigma_{x_{2}}^{4}{\left(\sigma_{T}^{2}+1\right)}^{2}\gamma_{004}^{*}),$ $U_{4}=\sigma_{y(0)}^{*4}-\theta \sigma_{y(0)}^{*2}\sigma_{T}^{2}{\bar{Z}}^{2}C_{Z}^{2},\;U_{5}=\theta \sigma_{x_{1}}^{2}(\sigma_{T}^{2}+1)(\sigma_{z}^{2}\gamma_{220}^{*}-2\sigma_{T}^{2}{\bar{Z}}^{2}C_{z}\gamma_{120}),$ $U_{6}=\theta \sigma_{x_{2}}^{2}(\sigma_{T}^{2}+1)(\sigma_{z}^{2}\gamma_{202}^{*}-2\sigma_{T}^{2}{\bar{Z}}^{2}C_{z}\gamma_{102}),\;U_{7}=\theta \sigma_{x_{1}}^{2}\sigma_{x_{2}}^{2}{\left(\sigma_{T}^{2}+1\right)}^{2}\gamma_{022}^{*}.$

From (27), the optimum values of *J*_*i*_(*i* = 1,2,3) are: $J_{1(opt)}=\frac{U_{4}(U_{2}U_{3}-U_{7}^{2})}{U_{1}U_{2}U_{3}-U_{1}U_{7}^{2}-U_{2}U_{6}^{2}-U_{3}U_{5}^{2}+2U_{5}U_{6}U_{7}},$ $J_{2(opt)}=\frac{U_{4}(U_{3}U_{5}-U_{6}U_{7})}{U_{1}U_{2}U_{3}-U_{1}U_{7}^{2}-U_{2}U_{6}^{2}-U_{3}U_{5}^{2}+2U_{5}U_{6}U_{7}},$ $J_{3(opt)}=\frac{\left(U_{2}U_{6}-U_{5}U_{7}\right)}{U_{1}U_{2}U_{3}-U_{1}U_{7}^{2}-U_{2}U_{6}^{2}-U_{3}U_{5}^{2}+2U_{5}U_{6}U_{7}}.$

Substituting the optimum values of *J*_*i*_(*i* = 1,2,3) in (27), we get minimum *MSE* of ${\hat{\sigma }}_{D_{2}}^{2}$ as given by

$$MSE{\left({\hat{\sigma }}_{D_{2}}^{2}\right)}_{\min}\approx \frac{\theta }{{\left(\sigma_{T}^{2}+1\right)}^{2}}[\sigma_{y}^{*4}-\frac{U_{4}^{2}(U_{2}U_{3}-U_{7}^{2})}{U_{1}(U_{2}U_{3}-U_{7}^{2})-U_{2}U_{6}^{2}-U_{3}U_{5}^{2}+2U_{5}U_{6}U_{7}}].$$ (28)

## 3. Saleem et al. estimator

Saleem et al. [20] suggested an estimator under Diana and Perri [8] model and proposed a new model also.

### (i) Saleem et al. [20] proposed estimator under Diana and Perri [8] Model

Recently Saleem et al. [20] suggested the following improved estimator for finite population variance, as given by:

$${\hat{\sigma }}_{I}^{2}=[\frac{K_{1}}{\sigma_{T}^{2}+1}\{s_{z}^{2}-\sigma_{S}^{2}-\sigma_{T}^{2}{\bar{z}}^{2}\}+K_{2}(\sigma_{x_{1}}^{2}-s_{x_{1}}^{2})+K_{3}(\sigma_{x_{2}}^{2}-s_{x_{2}}^{2})]$$

$$[{\left\{\exp(\frac{\sigma_{x_{1}}^{2}-s_{x_{1}}^{2}}{\sigma_{x_{1}}^{2}+s_{x_{2}}^{2}})\right\}}^{\lambda_{1}}{\left\{\exp(\frac{\sigma_{x_{2}}^{2}-s_{x_{2}}^{2}}{\sigma_{x_{2}}^{2}+s_{x_{2}}^{2}})\right\}}^{\lambda_{2}}],$$ (29)

where *K*_*i*_(*i* = 1,2,3) are constants and *λ*_*i*_(*i* = 1,2) are scaler quantities.

The bias and *MSE* expressions reported by Saleem et al. [20], are given by:

$$Bias({\hat{\sigma }}_{I}^{2})\approx \sigma_{y}^{2}(K_{1}-1)-K_{1}\sigma_{y}^{2}\theta \frac{1}{2}I_{1}+K_{1}\sigma_{z}^{2}\theta \frac{1}{2(\sigma_{T}^{2}+1)}I_{2}-K_{1}{\bar{Z}}^{2}\sigma_{T}^{2}C_{z}\theta \frac{1}{\left(\sigma_{T}^{2}+1\right)}I_{3}$$

$$-K_{2}\sigma_{x_{1}}^{2}\theta I_{4}-K_{3}\sigma_{x_{2}}^{2}\theta I_{5},$$ (30)

where $I_{1}=\lambda_{1}\{\lambda_{2}\gamma_{220}^{*}-\frac{1}{4}\lambda_{1}\gamma_{040}^{*}\}-\frac{1}{2}\lambda_{2}(\lambda_{2}-1)\gamma_{004}^{*},$ $I_{2}=\lambda_{1}\gamma_{220}^{*}-2\lambda_{2}\gamma_{202}^{*},$ $I_{3}=C_{z}+\lambda_{1}\gamma_{120}-2\lambda_{2}\gamma_{120},$ $I_{4}=\frac{1}{4}\lambda_{1}\gamma_{040}^{*}-\lambda_{2}\gamma_{022}^{*},$ $I_{5}=\frac{1}{2}\lambda_{1}\gamma_{022}^{*}-\lambda_{2}\gamma_{004}^{*},$ and

$$MSE{\left({\hat{\sigma }}_{I}^{2}\right)}_{\min}\approx \sigma_{y}^{6}(1-\frac{1}{A_{7}}),$$ (31)

where

$$A_{7}=\sigma_{y}^{4}+\theta A_{1}+\theta \gamma_{040}^{*}{\left(\frac{A_{4}}{A_{6}}\right)}^{2}+\theta \gamma_{004}^{*}{\left(\frac{A_{5}}{A_{6}}\right)}^{2}+2\theta \frac{A_{2}A_{4}}{A_{6}^{2}}-2\theta \frac{A_{3}A_{5}}{A_{6}}+2\theta \gamma_{022}^{*}\frac{A_{4}A_{5}}{A_{6}^{2}},$$

$$A_{1}=\sigma_{y}^{4}[\frac{1}{4}\lambda_{1}^{2}\gamma_{040}^{*}+\lambda_{2}^{2}\gamma_{004}^{*}-\lambda_{1}\lambda_{2}\gamma_{022}^{*}]+\frac{\sigma_{z}^{2}\gamma_{400}^{*}}{{\left(\sigma_{T}^{2}+1\right)}^{2}}+\frac{4{\bar{Z}}^{4}\sigma_{T}^{4}C_{z}^{2}}{{\left(\sigma_{T}^{2}+1\right)}^{2}}+\frac{{\bar{Z}}^{2}\sigma_{T}^{2}\gamma_{300}C_{z}\sigma_{z}^{2}}{\left(\sigma_{T}^{2}+1\right)}$$

$$-2\frac{\sigma_{y}^{2}}{\left(\sigma_{T}^{2}+1\right)}[\sigma_{z}^{2}\{\frac{1}{2}\lambda_{1}\gamma_{220}^{*}-\lambda_{2}\gamma_{202}^{*}\}+2{\bar{Z}}^{2}\sigma_{T}^{2}C_{z}\{\frac{1}{2}\lambda_{1}\gamma_{120}-\lambda_{2}\gamma_{102}\}],$$

$$A_{2}=\sigma_{y}^{2}[\frac{1}{2}\lambda_{1}\gamma_{040}^{*}-\lambda_{2}\gamma_{022}^{*}]-\frac{\sigma_{z}^{2}\gamma_{220}^{*}}{\left(\sigma_{T}^{2}+1\right)}+\frac{2{\bar{Z}}^{2}\sigma_{T}^{2}\gamma_{120}C_{z}}{\left(\sigma_{T}^{2}+1\right)},$$

$$A_{3}=\sigma_{y}^{2}[\frac{1}{2}\lambda_{1}\gamma_{022}^{*}-\lambda_{2}\gamma_{004}^{*}]-\frac{\sigma_{z}^{2}\gamma_{202}^{*}}{\left(\sigma_{T}^{2}+1\right)}+\frac{2{\bar{Z}}^{2}\sigma_{T}^{2}\gamma_{120}C_{z}}{\left(\sigma_{T}^{2}+1\right)},$$

$$A_{4}=A_{2}\gamma_{004}^{*}+A_{3}\gamma_{022}^{*},\;A_{5}=A_{2}\gamma_{022}^{*}+A_{3}\gamma_{040}^{*},\;A_{6}=\gamma_{040}^{*}\gamma_{004}^{*}-\gamma_{022}^{*2}.$$

The optimum values are: $K_{1opt}=\frac{\sigma_{y}^{2}}{A_{7}},$ $K_{2opt}=-\frac{K_{1}A_{4}}{\sigma_{x_{1}}^{2}A_{6}},$ $K_{3opt}=\frac{K_{1}A_{5}}{\sigma_{x_{2}}^{2}A_{6}}.$

**Note: It is observed that the bias and MSE expressions suggested by Saleem et al. (2023) are incorrect**.

### (ii) Saleem et al. [20] estimator under proposed RRT model

Saleem et al. [20] proposed the following RRT model and used it for obtaining bias and *MSE*.

$$Z_{I}=g(Y+\alpha S)+(1-g)T(Y+\alpha S),$$ (32)

where *Y* is the sensitive variable; *g* and *α* are the constants; *T* and *S* are two scrambled variables and are mutually uncorrelated with *Y* such that *E*(*S*) = 0 and *E*(*T*) = 1, $E(S^{2})=\sigma_{S}^{2}$ and $E(T^{2})=\sigma_{T}^{2}+1.$

$$\sigma_{Z_{I}}^{2}=\sigma_{y}^{2}G+\alpha^{2}\sigma_{S}^{2}G+{\left(1-g\right)}^{2}\sigma_{T}^{2}\mu_{Y}^{2},$$ (33)

where $G=g^{2}+(1-g^{2})(\sigma_{T}^{2}+1)$ and using $\mu_{y}=\bar{Z}$, we can write $\sigma_{y}^{2}$ as$\sigma_{y(I)}^{2}$:

$$\sigma_{y(I)}^{2}=\frac{1}{G}[\sigma_{Z_{I}}^{2}-{\left(1-g\right)}^{2}\sigma_{T}^{2}{\bar{Z}}^{2}]-\alpha^{2}\sigma_{S}^{2},$$ (34)

Estimating $\sigma_{Z_{I}}^{2}$ and ${\bar{Z}}^{2}$ by an unbiased estimator $s_{Z}^{2}$ and ${\bar{z}}^{2}$, we have

$${\hat{\sigma }}_{y(I)}^{2}=\frac{1}{G}[s_{Z}^{2}-\alpha^{2}\sigma_{S}^{2}G-{\left(1-g\right)}^{2}\sigma_{T}^{2}{\bar{z}}^{2}].$$ (35)

**Note: Saleem et al. [20] reported the expressions given in (33)–(35) are not correct.** The correct version is given below.

Rewriting (32) as given by:

$$Z_{I(P)}=g(Y+\alpha S)+(1-g)T(Y+\alpha S),$$ (36)

Solving the above expression given in (36), we have

$$\sigma_{Z_{I(P)}}^{2}=\sigma_{y}^{2}G+\alpha^{2}\sigma_{S}^{2}G+(G-1)\mu_{Y}^{2},$$ (37)

where *G* is defined above.

From (37), we have

$\sigma_{y(1)}^{2}=\frac{1}{G}[\sigma_{Z_{I(P)}}^{2}-\alpha^{2}\sigma_{S}^{2}G-(G-1){\bar{Z}}^{2}]$ as $\bar{Z}=\mu_{Y}$

Estimating $\sigma_{Z_{I(P)}}^{2}$ and ${\bar{Z}}^{2}$ by their unbiased estimators, we have

$${\hat{\sigma }}_{I(P)}^{2}=\frac{1}{G}[s_{z}^{2}-\alpha^{2}\sigma_{S}^{2}G-(G-1){\bar{z}}^{2}].$$ (38)

Note: Saleem et al. [20] used the proposed model given in (35) in their proposed estimator, but their resultant bias and *MSE* expressions are not correct.

## 4. Generalized RRM and suggested estimator

We use the generalized RRM in Saleem et al. [20] suggested estimator.

### (i) Generalized RRM

On the lines of Saleem et al. [20], a generalized RRM, is given by:

$$Z_{P}=h(Y+\alpha S)+(1-h)(TY+\alpha S),$$ (39)

where *Y* is the sensitive variable; *h* and *α* are the constants; *T* and *S* are two scrambled variables and are mutually uncorrelated with *Y* such that *E*(*S*) = 0 and *E*(*T*) = 1, $E(S^{2})=\sigma_{S}^{2}$ and $E(T^{2})=\sigma_{T}^{2}+1.$ By changing the values of *h* and *α*, we can obtain the additive and substrative types models.

Solving (39), we get

$$\sigma_{Z_{P}}^{2}=\sigma_{y}^{2}H+\alpha^{2}\sigma_{S}^{2}H^{*}+\mu_{Y}^{2}H^{**}),$$ (40)

where $H=h^{2}+(1-h^{2})(\sigma_{T}^{2}+1),$ $H^{*}=h^{2}+(1-h^{2}),$ and $H^{**}=h^{2}+(1-h^{2})(\sigma_{T}^{2}+1)-1.$ Using $\mu_{y}=\bar{Z}$, we can write $\sigma_{y}^{2}$ as $\sigma_{y(P)}^{2}$:

$$\sigma_{y(P)}^{2}=\frac{1}{H}[\sigma_{Z_{P}}^{2}-\alpha^{2}\sigma_{S}^{2}H^{*}-{\bar{Z}}^{2}H^{**}],\;as\;\bar{Z}=\mu_{Y}.$$ (41)

Estimating $\sigma_{Z_{P}}^{2}$ and ${\bar{Z}}^{2}$ by an unbiased estimator $s_{Z}^{2}$ and ${\bar{z}}^{2}$, we have

$${\hat{\sigma }}_{y(P)}^{2}=\frac{1}{H}[s_{z}^{2}-\alpha^{2}\sigma_{S}^{2}H^{*}-{\bar{z}}^{2}H^{**}].$$ (42)

### (ii) Use of RRM in Saleem et al. [20] estimator

On the lines of Saleem et al. [20], we propose the following estimator by using the generalized RRT model given in (42), as given by:

$${\hat{\sigma }}_{I(P)}^{2}=[\frac{K_{1c}}{H}\{s_{z}^{2}-\alpha^{2}\sigma_{S}^{2}H^{*}-{\bar{z}}^{2}H^{**})+K_{2c}(\sigma_{x_{1}}^{2}-s_{x_{1}}^{2})+K_{3c}(\sigma_{x_{2}}^{2}-s_{x_{2}}^{2})]$$

$$[{\left\{\exp(\frac{\sigma_{x_{1}}^{2}-s_{x_{1}}^{2}}{\sigma_{x_{1}}^{2}+s_{x_{2}}^{2}})\right\}}^{\lambda_{1c}}{\left(\frac{\sigma_{x_{2}}^{2}}{s_{x_{2}}^{2}}\right)}^{\lambda_{2c}}],$$ (43)

where *K*_*i*_(*i* = 1*c*,2*c*,3*c*) are constants and *λ*_*i*_(*i* = 1*c*,2*c*) are scaler quantities.

$${\hat{\sigma }}_{I(P)}^{2}\approx [\frac{K_{1c}}{H}\{\sigma_{z}^{2}(1+\zeta_{z})-\alpha^{2}\sigma_{S}^{2}H^{*}-{\bar{Z}}^{2}{\left(1+\zeta_{z}^{*}\right)}^{2}H^{**}\}-K_{2c}\sigma_{x_{1}}^{2}\zeta_{x_{1}}-K_{3c}\sigma_{x_{2}}^{2}\zeta_{x_{2}}]$$

$$[1-\frac{1}{2}\lambda_{1c}\zeta_{x_{1}}-\lambda_{2c}\zeta_{x_{2}}+\alpha_{1}\zeta_{x_{1}}^{2}+\alpha_{2}\zeta_{x_{2}}^{2}+\alpha_{3}\zeta_{x_{1}}\zeta_{x_{2}}],$$ (44)

where $\alpha_{1}=\frac{1}{8}\lambda_{1c}(\lambda_{1c}+2),$ $\alpha_{2}=\frac{1}{2}\lambda_{2c}(\lambda_{2c}+1),$ and $\alpha_{3}=\frac{1}{2}\lambda_{1c}\lambda_{2c}.$ Solving (44), we get

$${\hat{\sigma }}_{I(P)}^{2}-\sigma_{y(P)}^{2}\approx \frac{1}{H}[(K_{1c}-1)\sigma_{y(P)}^{*2}+K_{1c}\{\sigma_{Z}^{2}(\zeta_{z}-\frac{1}{2}\lambda_{1c}\zeta_{x_{1}}-\lambda_{2c}\zeta_{x_{2}}-\frac{1}{2}\lambda_{1c}\zeta_{x_{1}}\zeta_{z}-\lambda_{2c}\zeta_{x_{2}}\zeta_{z}$$

$$+\alpha_{1}\zeta_{x_{1}}^{2}+\alpha_{2}\zeta_{x_{2}}^{2}+\alpha_{3}\zeta_{x_{1}}\zeta_{x_{2}}\}-\sigma_{S}^{2}\alpha^{2}H^{*}(-\frac{1}{2}\lambda_{1c}\zeta_{x_{1}}-\lambda_{2c}\zeta_{x_{2}}+\alpha_{1}\zeta_{x_{1}}^{2}+\alpha_{2}\zeta_{x_{2}}^{2}+\alpha_{3}\zeta_{x_{1}}\zeta_{x_{2}})$$

$$-{\bar{Z}}^{2}H^{**}(-\frac{1}{2}\lambda_{1c}\zeta_{x_{1}}-\lambda_{2c}\zeta_{x_{2}}+\zeta_{z}^{*2}-\lambda_{1c}\zeta_{x_{1}}\zeta_{z}^{*}-2\lambda_{2c}\zeta_{x_{2}}\zeta_{z}^{*}+2\zeta_{z}^{*}+\alpha_{1}\zeta_{x_{1}}^{2}+\alpha_{2}\zeta_{x_{2}}^{2}+\alpha_{3}\zeta_{x_{1}}\zeta_{x_{2}})\}$$

$$-K_{2c}H\sigma_{x_{1}}^{2}\{\zeta_{x_{1}}-\frac{1}{2}\lambda_{1c}\zeta_{x_{1}}^{2}-\lambda_{2c}\zeta_{x_{1}}\zeta_{x_{2}}\}-K_{3c}H\sigma_{x_{2}}^{2}\left\{\zeta_{x_{2}}-\lambda_{2c}\zeta_{x_{2}}^{2}-\frac{1}{2}\lambda_{1c}\zeta_{x_{1}}\zeta_{x_{2}}\right\}],$$ (45)

where $\sigma_{y(P)}^{2}=\frac{1}{H}\sigma_{y(P)}^{*2}$ and $\sigma_{y(P)}^{*2}=(\sigma_{z}^{2}-\alpha^{2}\sigma_{S}^{2}H^{*}-H^{**}{\bar{Z}}^{2}).$ From (45), the bias of ${\hat{\sigma }}_{I(P)}^{2}$ to first degree of approximation, is given by

$$Bias({\hat{\sigma }}_{I(P)}^{2})\approx \frac{1}{H}[(K_{1c}-1)\sigma_{y(P)}^{*2}+K_{1c}\{\theta \sigma_{Z}^{2}(-\frac{1}{2}\lambda_{1c}\gamma_{220}^{*}-\lambda_{2c}\gamma_{202}^{*}+\alpha_{1}\gamma_{040}^{*}+\alpha_{2}\gamma_{004}^{*}+\alpha_{3}\gamma_{022}^{*})$$

$$-\sigma_{S}^{2}\alpha^{2}H^{*}\theta (\alpha_{1}\gamma_{040}^{*}+\alpha_{2}\gamma_{004}^{*}+\alpha_{3}\gamma_{022}^{*})-{\bar{Z}}^{2}H^{**}(C_{z}^{2}-\lambda_{1c}\gamma_{120}C_{z}-2\lambda_{2c}\gamma_{102}C_{z}+\alpha_{1}\gamma_{040}^{*}+\alpha_{2}\gamma_{004}^{*}+\alpha_{3}\gamma_{022}^{*})\}$$

$$-K_{2c}H\sigma_{x_{1}}^{2}\theta \sigma_{x_{1}}^{2}\{-\frac{1}{2}\lambda_{1c}\gamma_{040}^{*}-\lambda_{2c}\gamma_{022}^{*}\}-K_{3c}H\sigma_{x_{2}}^{2}\theta \{-\frac{1}{2}\lambda_{1c}\gamma_{022}^{*}-\lambda_{2c}\gamma_{004}^{*}\}].$$ (46)

Squaring (45) and then taking expectation, we get the *MSE* of ${\hat{\sigma }}_{I(P)}^{2}$ and is given by:

$$MSE({\hat{\sigma }}_{y(P)}^{2})\approx \frac{1}{H^{2}}E[\sigma_{y(P)}^{*4}+K_{1c}^{2}\alpha_{1c}+K_{2c}^{2}\alpha_{2c}+K_{3c}^{2}\alpha_{3c}-2K_{1c}\alpha_{4c}+2K_{2c}\alpha_{5c}+2K_{3c}\alpha_{6c}$$

$$-2K_{1c}K_{2c}\alpha_{7c}-2K_{1c}K_{3c}\alpha_{8c}+2K_{2c}K_{3c}\alpha_{9c}],$$ (47)

where

$$\alpha_{1c}=\sigma_{y(P)}^{*4}+\{\sigma_{z}^{4}\theta (\gamma_{400}^{*}+\frac{1}{4}\lambda_{1c}^{2}\gamma_{040}^{*}+\lambda_{2c}^{2}\gamma_{004}^{*}-\lambda_{1c}\gamma_{220}^{*}-2\lambda_{2c}\gamma_{202}^{*}+\lambda_{1c}\lambda_{2c}\gamma_{022}^{*})$$

$$+\sigma_{S}^{4}\alpha^{4}H^{*2}\theta (\frac{1}{4}\lambda_{1c}^{2}\gamma_{040}^{*}+\lambda_{2c}^{2}\gamma_{004}^{*}+\lambda_{1c}\lambda_{2c}\gamma_{022}^{*})+{\bar{Z}}^{4}H^{**2}\theta (\frac{1}{4}\lambda_{1c}^{2}\gamma_{040}^{*}+\lambda_{2c}^{2}\gamma_{004}^{*}$$

$$+4C_{z}^{2}+\lambda_{1c}\lambda_{2c}\gamma_{022}^{*}-2\lambda_{1c}\gamma_{120}C_{z}-4\lambda_{2c}\gamma_{102}C_{z})-2\sigma_{z}^{4}\sigma_{S}^{2}\alpha^{2}H^{*}\theta (-\frac{1}{2}\lambda_{1c}\gamma_{220}^{*}-\lambda_{2c}\gamma_{202}^{*}$$

$$+\frac{1}{4}\lambda_{1c}^{2}\gamma_{040}^{*}+\lambda_{1c}\lambda_{2c}\gamma_{022}^{*}+\lambda_{2c}^{2}\gamma_{004}^{*})-2\sigma_{z}^{2}{\bar{Z}}^{2}H^{**}\theta (-\frac{1}{2}\lambda_{1c}\gamma_{220}^{*}-\lambda_{2c}\gamma_{202}^{*}+2\gamma_{300}C_{z}+\frac{1}{4}\lambda_{1c}^{2}\gamma_{040}^{*}$$

$$+\lambda_{1c}\lambda_{2c}\gamma_{022}^{*}-\lambda_{1c}\gamma_{120}C_{z}-2\lambda_{2c}\gamma_{102}C_{z}+\lambda_{2c}^{2}\gamma_{004}^{*})+2\sigma_{S}^{2}\alpha^{2}{\bar{Z}}^{2}H^{*}H^{**}\theta (\frac{1}{4}\lambda_{1c}^{2}\gamma_{040}^{*}+\lambda_{1c}\lambda_{2c}\gamma_{022}^{*}$$

$$\left(-\lambda_{1c}\gamma_{120}C_{z}+\lambda_{2c}^{2}\gamma_{004}^{*}-2\lambda_{2c}\gamma_{102}C_{z}\right)\}+2\sigma_{y(P)}^{*2}\{\sigma_{z}^{2}\theta (-\frac{1}{2}\lambda_{1c}\gamma_{220}^{*}-\lambda_{2c}\gamma_{202}^{*}+\alpha_{1}\gamma_{040}^{*}+\alpha_{2}\gamma_{004}^{*}+\alpha_{3}\gamma_{022}^{*})$$

$$-\sigma_{S}^{2}\alpha^{2}H^{*}\theta (\alpha_{1}\gamma_{040}^{*}+\alpha_{2}\gamma_{004}^{*}+\alpha_{3}\gamma_{022}^{*})-{\bar{Z}}^{2}H^{**}\theta \{\left(C_{z}^{2}-\lambda_{1c}\gamma_{120}C_{z}-2\lambda_{2c}\gamma_{102}C_{z}+\alpha_{1}\gamma_{040}^{*}+\alpha_{2}\gamma_{004}^{*}+\alpha_{3}\gamma_{022}^{*}\right)\},$$

$$\alpha_{2c}=H^{2}\theta \sigma_{x_{1}}^{4}\gamma_{040}^{*},\;\alpha_{3c}=H^{2}\theta \sigma_{x_{2}}^{4}\gamma_{004}^{*},$$

$$\alpha_{4c}=\sigma_{y(P)}^{*4}+\sigma_{y(P)}^{*2}\{\sigma_{z}^{2}\theta (-\frac{1}{2}\lambda_{1c}\gamma_{220}^{*}-\lambda_{2c}\gamma_{202}^{*}+\alpha_{1}\gamma_{040}^{*}+\alpha_{2}\gamma_{004}^{*}+\alpha_{3}\gamma_{022}^{*})$$

$$-\sigma_{S}^{2}\alpha^{2}H^{*}\theta (\alpha_{1}\gamma_{040}^{*}+\alpha_{2}\gamma_{004}^{*}+\alpha_{3}\gamma_{022}^{*})$$

$$-{\bar{Z}}^{2}H^{**}\theta \left(C_{z}^{2}-\lambda_{1c}\gamma_{120}C_{z}-2\lambda_{2c}\gamma_{102}C_{z}+\alpha_{1}\gamma_{040}^{*}+\alpha_{2}\gamma_{004}^{*}+\alpha_{3}\gamma_{022}^{*}\right)\},$$

$$\alpha_{5c}=\sigma_{y(P)}^{*2}H\theta \sigma_{x_{1}}^{2}(-\frac{1}{2}\lambda_{1c}\gamma_{040}^{*}-\lambda_{2c}\gamma_{022}^{*}),\;\alpha_{6c}=\sigma_{y(P)}^{*2}H\theta \sigma_{x_{2}}^{2}(-\frac{1}{2}\lambda_{1c}\gamma_{022}^{*}-\lambda_{2c}\gamma_{004}^{*}),$$

$$\alpha_{7c}=\sigma_{y(P)}^{*2}H\theta \sigma_{x_{1}}^{2}(-\frac{1}{2}\lambda_{1c}\gamma_{040}^{*}-\lambda_{2c}\gamma_{022}^{*})+H\theta \sigma_{x_{1}}^{2}\sigma_{z}^{2}(\gamma_{220}^{*}-\frac{1}{2}\lambda_{1c}\gamma_{040}^{*}-\lambda_{2c}\gamma_{022}^{*}),$$

$$\alpha_{8c}=H[\sigma_{y}^{*2}\theta \sigma_{x_{2}}^{2}(-\frac{1}{2}\lambda_{1c}\gamma_{022}^{*}-\lambda_{2c}\gamma_{004}^{*})+\theta \sigma_{x_{2}}^{2}\{\sigma_{z}^{2}(\gamma_{202}^{*}-\frac{1}{2}\lambda_{1c}\gamma_{022}^{*}-\lambda_{2c}\gamma_{004}^{*})$$

$$-\sigma_{S}^{2}\alpha^{2}H^{*}(\frac{1}{2}\lambda_{1c}\gamma_{022}^{*}-\lambda_{2c}\gamma_{004}^{*})-{\bar{Z}}^{2}H^{**}\{\left(-\frac{1}{2}\lambda_{1c}\gamma_{022}^{*}-\lambda_{2c}\gamma_{004}^{*}+2\gamma_{102}C_{z}\right)\}].$$

The minimum *MSE* of ${\hat{\sigma }}_{I(P)}^{2}$, is given by

$$MSE{\left({\hat{\sigma }}_{I(P)}^{2}\right)}_{\min}\approx \frac{1}{H^{2}}(\sigma_{y(P)}^{*4}+\frac{O_{5c}}{O_{1c}}).$$ (48)

The optimum values are:

$$K_{1c(opt)}=\frac{O_{2c}}{O_{1c}},\;K_{2c(opt)}=-\frac{O_{3c}}{O_{1c}},K_{3c(opt)}=-\frac{O_{4c}}{O_{1c}},$$

where

$$O_{1c}=\alpha_{1c}\alpha_{2c}\alpha_{3c}-\alpha_{1c}\alpha_{9c}^{2}-\alpha_{2c}\alpha_{8c}^{2}-\alpha_{3c}\alpha_{7c}^{2}+2\alpha_{7c}\alpha_{8c}\alpha_{9c},$$

$$O_{2c}=\alpha_{2c}\alpha_{3c}\alpha_{4c}-\alpha_{2c}\alpha_{6c}\alpha_{8c}-\alpha_{3c}\alpha_{5c}\alpha_{7c}-\alpha_{4c}\alpha_{9c}^{2}+\alpha_{5c}\alpha_{8c}\alpha_{9c}+\alpha_{6c}\alpha_{7c}\alpha_{9c},$$

$$O_{3c}=\alpha_{1c}\alpha_{3c}\alpha_{5c}-\alpha_{1c}\alpha_{6c}\alpha_{9c}-\alpha_{3c}\alpha_{4c}\alpha_{7c}+\alpha_{4c}\alpha_{8c}\alpha_{9c}-\alpha_{5c}\alpha_{8c}^{2}+\alpha_{6c}\alpha_{7c}\alpha_{9c},$$

$$O_{4c}=\alpha_{1c}\alpha_{2c}\alpha_{6c}-\alpha_{1c}\alpha_{5c}\alpha_{9c}-\alpha_{2c}\alpha_{4c}\alpha_{8c}+\alpha_{4c}\alpha_{7c}\alpha_{9c}+\alpha_{5c}\alpha_{7c}\alpha_{8c}-\alpha_{6c}\alpha_{7c}^{2},$$

$$O_{5c}=m_{1}\alpha_{4c}^{2}+(m_{2}\alpha_{5c}+2m_{3}\alpha_{6c})\alpha_{4c}+m_{4}\alpha_{5c}^{2}+2m_{5}\alpha_{5c}\alpha_{6c}-m_{6}\alpha_{6c}^{2},$$

$$m_{1c}=\alpha_{9c}^{2}-\alpha_{2c}\alpha_{3c},\;m_{2c}=2\alpha_{3c}\alpha_{7c}-2\alpha_{8c}\alpha_{9c},\;m_{3c}=\alpha_{2c}\alpha_{8c}-\alpha_{7c}\alpha_{9c},\;m_{4c}=\alpha_{8c}^{2}-\alpha_{1c}\alpha_{3c},m_{5c}=\alpha_{1c}\alpha_{9c}-\alpha_{7c}\alpha_{8c},\;m_{6c}=\alpha_{1c}\alpha_{2c}-\alpha_{7c}^{2}.$$

## 5. Azeem et al. [6] RRT model

Azeem et al. [6] suggested the following RRT model to obtain the bias and *MSE* of the usual variance estimator and the ratio estimator.

$$Z_{A}=l(Y+S)+(1-l)(Y+YS),$$ (49)

where *Y* is the sensitive variable; 0≤*l*≤1; *S* is scrambled variables and are mutually uncorrelated with *Y* such that *E*(*S*) = 0, $E(S^{2})=\sigma_{S}^{2}$, *E*(*Y*) = *μ*_*Y*_, and $E(Y^{2})=\mu_{Y}^{2}+\sigma_{Y}^{2}$.

$$\sigma_{z_{A}}^{2}=A\sigma_{y}^{2}+l^{2}\sigma_{S}^{2}+{\left(1-l\right)}^{2}\sigma_{S}^{2}\mu_{Y}^{2},$$ (50)

where $A=l^{2}+(1-l^{2})(1+\sigma_{S}^{2})$ and using $\mu_{y}=\bar{Z}$, we can write $\sigma_{y}^{2}$ as $\sigma_{y(A)}^{2}$:

$$\sigma_{y(A)}^{2}=\frac{1}{A}[\sigma_{z_{A}}^{2}-l^{2}\sigma_{S}^{2}+(1-l{)}^{2}\sigma_{S}^{2}{\bar{Z}}^{2}].$$ (51)

Estimating $\sigma_{Z_{A}}^{2}$ and ${\bar{Z}}^{2}$ by an unbiased estimator $s_{Z}^{2}$ and ${\bar{z}}^{2}$, we have

$${\hat{\sigma }}_{y(A)}^{2}=\frac{1}{A}[s_{Z}^{2}-l^{2}\sigma_{S}^{2}+{\left(1-l\right)}^{2}\sigma_{S}^{2}{\bar{z}}^{2}],$$ (52)

**Note:** It is observed that the expressions given in (50)–(52) by Azeem et al. [6] are not correct. So bias and *MSE* expressions based on the estimator ${\hat{\sigma }}_{y(A)}^{2}$ and consequently numerical results may be misleading.

The correct expression of the Azeem et al. [6] model, is given by

$$\sigma_{z_{(A)C}}^{2}=A\sigma_{y}^{2}+l^{2}\sigma_{S}^{2}+{\left(A-1\right)}^{2}\mu_{Y}^{2},$$ (53)

Using $\mu_{y}=\bar{Z}$, we can write $\sigma_{y}^{2}$ as $\sigma_{y(A)C}^{2}$:

$$\sigma_{y(A)C}^{2}=\frac{1}{A}[\sigma_{z_{(A)C}}^{2}-l^{2}\sigma_{S}^{2}-(A-1){\bar{Z}}^{2}],$$ (54)

Estimating $\sigma_{Z_{A(C)}}^{2}$ and ${\bar{Z}}^{2}$ by an unbiased estimator $s_{Z}^{2}$ and ${\bar{z}}^{2}$, we have

$${\hat{\sigma }}_{y(A)C}^{2}=\frac{1}{A}[s_{Z}^{2}-l^{2}\sigma_{S}^{2}-(A-1){\bar{z}}^{2}],$$ (55)

The bias and *MSE* of ${\hat{\sigma }}_{y(A)C}^{2}$ to first order of approximation, are given by

$$Bias({\hat{\sigma }}_{y(A)C}^{2})\approx -\frac{1}{A}\theta {\bar{Z}}^{2}C_{z}^{2}(A-1)$$ (56)

and

$$MSE({\hat{\sigma }}_{y(A)C}^{2})\approx -\frac{1}{A^{2}}\theta [\sigma_{z}^{4}\lambda_{400}^{*}+4{\bar{Z}}^{4}{\left(A-1\right)}^{2}C_{z}^{2}-4\sigma_{z}^{2}{\bar{Z}}^{2}(A-1)C_{z}\lambda_{300}].$$ (57)

Azeem et al. [6] suggested the following ratio estimator

$${\hat{\sigma }}_{R(A)}^{2}={\hat{\sigma }}_{y(A)}^{2}(\frac{s_{x}^{*2}}{\sigma_{x}^{2}}),$$ (58)

where $s_{x}^{*2}=\frac{(N-1)\sigma_{x}^{2}-(n-1)s_{x}^{2}}{N-n}$.

The bias and *MSE* of ${\hat{\sigma }}_{R(A)}^{2}$ are also not correct (For detail, see Azeem et al. [6])

The ratio estimator based on correct model, is given by

$${\hat{\sigma }}_{R(A)C}^{2}={\hat{\sigma }}_{y(A)C}^{2}(\frac{s_{x}^{*2}}{\sigma_{x}^{2}}),$$ (59)

The bias and *MSE* of ${\hat{\sigma }}_{R(A)C}^{2}$ to first order of approximation, are given by

$$Bias({\hat{\sigma }}_{R(A)C}^{2})\approx -\frac{\theta }{A}[D\sigma_{z}^{2}\lambda_{220}^{*}+{\bar{Z}}^{2}(A-1)(C_{z}^{2}-2D\lambda_{120}C_{z})],$$ (60)

where $D=\frac{n-1}{N-n}$ and

$$MSE({\hat{\sigma }}_{R(A)C}^{2})\approx \frac{1}{A^{2}}\left[\sigma_{z}^{4}(\lambda_{400}^{*}+D^{2}\lambda_{040}^{*}-2D\lambda_{220}^{*}\right))+{\bar{Z}}^{4}(4C_{z}^{2}+D^{2}\lambda_{040}^{*}-4D\lambda_{120}C_{z})$$

$$+l^{4}\sigma_{S}^{2}D^{2}\lambda_{040}^{*}-2\sigma_{z}^{2}{\bar{Z}}^{2}(2\lambda_{300}C_{z}-D\lambda_{220}^{*}-2D\lambda_{120}C_{z}+D^{2}\lambda_{040}^{*})$$

$$+2\sigma_{z}^{2}l^{2}D^{2}\sigma_{S}^{2}D(\lambda_{220}^{*}-D\lambda_{040}^{*})-2\sigma_{S}^{2}{\bar{Z}}^{2}l^{2}D(A-1)(2\lambda_{120}C_{z}-D^{2}\lambda_{040}^{*})].$$ (61)

## 6. Proposed estimator

The purpose of the suggested estimator is to construct such a type of estimator which should provide better estimation as compared to all other previously considered estimators. On the lines of Gupta et al. [10] and Saleem et al. [20], and Azeem et al. [6], we propose the following generalized class of difference-in-exponential ratio-product type estimators for finite population variance as given by:

$${\hat{\sigma }}_{P}^{2}=[\frac{L_{1}}{H}\{s_{z}^{2}-\alpha^{2}\sigma_{S}^{2}H^{*}-{\bar{z}}^{2}H^{**}\}{\left\{\exp(\frac{\sigma_{x_{1}}^{2}-s_{x_{1}}^{2}}{\sigma_{x_{1}}^{2}+s_{x_{1}}^{2}})\right\}}^{\pi_{1}}]$$

$$+L_{2}{\left\{\exp(\frac{s_{x_{1}}^{2}-\sigma_{x_{1}}^{2}}{s_{x_{1}}^{2}+\sigma_{x_{1}}^{2}})\right\}}^{\pi_{2}}+L_{3}{\left\{\exp(\frac{s_{x_{2}}^{2}-\sigma_{x_{2}}^{2}}{s_{x_{2}}^{2}+\sigma_{x_{2}}^{2}})\right\}}^{\pi_{3}},$$ (62)

where *L*_*i*_(*i* = 1,2,3) are constants, whose values are to be determined and *π*_*i*_(*i* = 1,2,3) are scalar quantities.

In terms of errors, we have

$${\hat{\sigma }}_{P}^{2}\approx \frac{L_{1}}{G}[\sigma_{z}^{2}(1+\zeta_{z})-\alpha^{2}\sigma_{S}^{2}H^{*}-{\bar{Z}}^{2}H^{**}{\left(1+\zeta_{z}^{*}\right)}^{2}][1-\frac{1}{2}\pi_{1}\zeta_{x_{1}}+\frac{1}{8}\pi_{1}(\pi_{1}+2)\zeta_{x_{1}}^{2}]$$

$$+L_{2}[1+\frac{1}{2}\pi_{2}\zeta_{x_{1}}+\frac{1}{8}\pi_{2}(\pi_{2}-2)\zeta_{x_{1}}^{2}]+L_{3}[1+\frac{1}{2}\pi_{3}\zeta_{x_{2}}+\frac{1}{8}\pi_{3}(\pi_{3}-2)\zeta_{x_{2}}^{2}]$$

or

$${\hat{\sigma }}_{P}^{2}-\sigma_{y(P)}^{*2}\approx \frac{1}{H}[(L_{1}-1)\sigma_{y(P)}^{*2}+L_{1}\{\sigma_{Z}^{2}(\zeta_{z}-\frac{1}{2}\pi_{1}\zeta_{x_{1}}-\frac{1}{2}\pi_{1}\zeta_{x_{1}}\zeta_{z}+\frac{1}{8}\pi_{1}(\pi_{1}+2)\zeta_{x_{1}}^{2})$$

$$-\sigma_{S}^{2}\alpha^{2}H^{*}(-\frac{1}{2}\pi_{1}\zeta_{x_{1}}+\frac{1}{8}\pi_{1}(\pi_{1}+2)\zeta_{x_{1}}^{2})$$

$$-{\bar{Z}}^{2}H^{**}(-\frac{1}{2}\pi_{1}\zeta_{x_{1}}+2\zeta_{z}^{*}+\zeta_{z}^{*2}-\pi_{1}\zeta_{x_{1}}\zeta_{z}^{*}+\frac{1}{8}\pi_{1}(\pi_{1}+2)\zeta_{x_{1}}^{2})\}]$$

$$+L_{2}H\{1+\frac{1}{2}L_{2}\zeta_{x_{1}}+\frac{1}{8}\pi_{2}(\pi_{2}-2)\zeta_{x_{1}}^{2}\}+L_{3}H\{1+\frac{1}{2}L_{2}\zeta_{x_{2}}+\frac{1}{8}\pi_{3}(\pi_{3}-2)\zeta_{x_{2}}^{2}\},$$ (63)

where $\sigma_{y(P)}^{*2}=(\sigma_{z}^{2}-\alpha^{2}\sigma_{S}^{2}H^{*}-{\bar{Z}}^{2}H^{**})$.

From (63), the bias of ${\hat{\sigma }}_{P}^{2}$ to first order of approximation, is given by

$$Bias({\hat{\sigma }}_{P}^{2})\approx \frac{1}{H}[(L_{1}-1)\sigma_{y(P)}^{*2}+L_{1}\theta \{\sigma_{z}^{2}(-\frac{1}{2}\pi_{1}\gamma_{220}^{*}+\frac{1}{8}(\pi_{1}+2)\gamma_{040}^{*})$$

$$-\alpha^{2}\sigma_{S}^{2}H^{*}(\frac{1}{8}\pi_{1}(\pi_{1}+2)\gamma_{040}^{*})-{\bar{Z}}^{2}H^{**}(C_{z}^{2}-\pi_{1}\gamma_{120}C_{z}+\frac{1}{8}\pi_{1}(\pi_{1}+2)\gamma_{040}^{*})\}$$

$$+L_{2}H\{1+\frac{1}{8}\theta \pi_{2}(\pi_{2}-2)\gamma_{040}^{*}\}+L_{3}H\{1+\frac{1}{8}\theta \pi_{2}(\pi_{2}-2)\gamma_{004}^{*}\}].$$ (64)

From (63), *MSE* of ${\hat{\sigma }}_{P}^{2}$ to first order of approximation, is given by

$$MSE({\hat{\sigma }}_{P}^{2})\approx \frac{1}{H^{2}}[\sigma_{y(P)}^{*4}+L_{1}^{2}\eta_{1}+L_{2}^{2}\eta_{2}+L_{3}^{2}\eta_{3}-2L_{1}\eta_{4}-2L_{2}\eta_{5}-2L_{3}\eta_{6}$$

$$+2L_{1}L_{2}\eta_{7}+2L_{1}L_{3}\eta_{8}+2L_{2}L_{3}\eta_{9}],$$ (65)

where

$$\eta_{1}=\sigma_{y(P)}^{*4}+\sigma_{z}^{4}\theta (\gamma_{400}^{*}+\frac{1}{4}\pi_{1}^{2}\gamma_{040}^{*}-\pi_{1}\gamma_{220}^{*})+\frac{1}{4}\sigma_{S}^{4}\theta \alpha^{4}H^{*2}\pi_{1}^{2}\gamma_{040}^{*}$$

$$-2\alpha^{2}\sigma_{S}^{2}\sigma_{z}^{2}H^{*}\theta (-\frac{1}{2}\pi_{1}\gamma_{220}^{*}+\frac{1}{4}\pi_{1}^{2}\gamma_{040}^{*})+{\bar{Z}}^{4}H^{**2}\theta (4C_{z}^{2}+\frac{1}{4}\pi_{1}^{2}\gamma_{040}^{*}-2\pi_{1}\gamma_{120}C_{z})$$

$$-2{\bar{Z}}^{2}\sigma_{z}^{2}H^{**}\theta (-\frac{1}{2}\pi_{1}\gamma_{220}^{*}+2\gamma_{300}C_{z}+\frac{1}{4}\pi_{1}^{2}\gamma_{040}^{*}-\pi_{1}\gamma_{120}C_{z})$$

$$+2\alpha^{2}\sigma_{S}^{2}\sigma_{T}^{2}{\bar{Z}}^{2}G^{*}G^{**}\theta (\frac{1}{4}\pi_{1}^{2}\gamma_{040}^{*}-\pi_{1}\gamma_{120}C_{z})]+2\sigma_{y(P)}^{*2}\left[\sigma_{z}^{2}\theta \{-\frac{1}{2}\pi_{1}\gamma_{220}^{*}+\frac{1}{8}\pi_{1}(\pi_{1}+2)\gamma_{040}^{*}\right\}$$

$$-\alpha^{2}\sigma_{S}^{2}H^{*}\theta (\frac{1}{8}\pi_{1}(\pi_{1}+2)\gamma_{040}^{*})-{\bar{Z}}^{2}H^{**}\theta \{C_{z}^{2}-\pi_{1}\gamma_{120}C_{z}+\frac{1}{8}\pi_{1}(\pi_{1}+2)\gamma_{040}^{*}\}],$$

$$\eta_{2}=H^{2}\{1+\frac{1}{2}\theta \pi_{2}(\pi_{2}-1)\gamma_{040}^{*}\},$$

$$\eta_{3}=H^{2}\{1+\frac{1}{2}\theta \pi_{3}(\pi_{3}-1)\gamma_{004}^{*}\},$$

$$\eta_{4}=\sigma_{y(P)}^{*4}+\sigma_{y(P)}^{*2}\theta [\sigma_{z}^{2}\{-\frac{1}{2}\pi_{1}\gamma_{220}^{*}+\frac{1}{8}\pi_{1}(\pi_{1}+2)\gamma_{040}^{*}\}-\alpha^{2}\sigma_{S}^{2}H^{*}\frac{1}{8}\pi_{1}(\pi_{1}+2)\gamma_{040}^{*}$$

$$-{\bar{Z}}^{2}H^{**}\{C_{z}^{2}-\pi_{1}\gamma_{120}C_{z}+\frac{1}{8}\pi_{1}(\pi_{1}+2)\gamma_{040}^{*}\}],$$

$$\eta_{5}=\sigma_{y(P)}^{*2}H\{1+\frac{1}{8}\theta \pi_{2}(\pi_{2}-2)\gamma_{040}^{*}\},$$

$$\eta_{6}=\sigma_{y(P)}^{*2}H\{1+\frac{1}{8}\theta \pi_{3}(\pi_{3}-2)\gamma_{004}^{*}\},$$

$$\eta_{7}=H[\sigma_{y(P)}^{*2}(1+\frac{1}{8}\theta \pi_{2}(\pi_{2}-1)\gamma_{040}^{*})+\sigma_{z}^{2}\theta (\frac{1}{2}(\pi_{2}-\pi_{1})\gamma_{220}^{*}+\pi_{1}(\pi_{1}-2\pi_{2}+2)\gamma_{040}^{*})$$

$$-\alpha^{2}\sigma_{S}^{2}H^{*}\theta (\frac{1}{8}\pi_{1}(\pi_{1}-2\pi_{2}+2)\gamma_{040}^{*})-{\bar{Z}}^{2}H^{**}\theta (C_{z}^{2}+(\pi_{2}-\pi_{1})\gamma_{120}C_{z}+\frac{1}{8}\pi_{1}(\pi_{1}-2\pi_{2}+2)\gamma_{040}^{*})],$$

$$\eta_{8}=H[\sigma_{y(P)}^{*2}(1+\frac{1}{8}\theta \pi_{3}(\pi_{3}-1)\gamma_{004}^{*})+\sigma_{z}^{2}\theta (\frac{1}{2}\pi_{3}\gamma_{202}^{*}-\frac{1}{4}\pi_{1}\pi_{3}\gamma_{022}^{*}+\frac{1}{8}\pi_{1}(\pi_{1}+2)\gamma_{040}^{*}-\frac{1}{2}\pi_{1}\gamma_{220}^{*})$$

$$-\alpha^{2}\sigma_{S}^{2}H^{*}\theta (-\frac{1}{4}\pi_{1}\pi_{3}\gamma_{022}^{*}+\frac{1}{8}\pi_{1}(\pi_{1}+2)\gamma_{040}^{*})$$

$$-{\bar{Z}}^{2}H^{**}\theta (-\frac{1}{4}\pi_{1}\pi_{3}\gamma_{022}^{*}+\pi_{3}\gamma_{102}C_{z}+C_{z}^{2}+\frac{1}{8}\pi_{1}(\pi_{1}+2)\gamma_{040}^{*})],$$

$$\eta_{9}=H^{2}(1+\frac{1}{4}\theta \pi_{2}\pi_{3}\gamma_{022}^{*}+\frac{1}{8}\pi_{2}(\pi_{2}-2)\gamma_{040}^{*}+\frac{1}{8}\pi_{3}(\pi_{3}-2)\gamma_{004}^{*})].$$

The minimum *MSE* of ${\hat{\sigma }}_{P}^{2}$, is given by

$$MSE{\left({\hat{\sigma }}_{P}^{2}\right)}_{\min}\approx \frac{1}{H^{2}}[\sigma_{y(P)}^{*4}+\frac{Q_{5}}{Q_{1}}].$$ (66)

The optimum values *L*_*i*_(*i* = 1,2,3) are given by $L_{1(opt)}=\frac{Q_{2}}{Q_{1}},$ $L_{2(opt)}=\frac{Q_{3}}{Q_{1}},$ $L_{3(opt)}=\frac{Q_{4}}{Q_{1}},$ where

$$Q_{1}=\eta_{1}\eta_{2}\eta_{3}-\eta_{1}\eta_{9}^{2}-\eta_{2}\eta_{8}^{2}-\eta_{3}\eta_{7}^{2}+2\eta_{7}\eta_{8}\eta_{9},$$

$$Q_{2}=\eta_{2}\eta_{3}\eta_{4}-\eta_{2}\eta_{6}\eta_{8}-\eta_{3}\eta_{5}\eta_{7}-\eta_{4}\eta_{9}^{2}+\eta_{5}\eta_{8}\eta_{9}+\eta_{6}\eta_{7}\eta_{9},$$

$$Q_{3}=\eta_{1}\eta_{3}\eta_{5}-\eta_{1}\eta_{6}\eta_{9}-\eta_{3}\eta_{4}\eta_{7}+\eta_{4}\eta_{8}\eta_{9}-\eta_{5}\eta_{8}^{2}+\eta_{6}\eta_{7}\eta_{9},$$

$$Q_{4}=\eta_{1}\eta_{2}\eta_{6}-\eta_{1}\eta_{5}\eta_{9}-\eta_{2}\eta_{4}\eta_{8}+\eta_{4}\eta_{7}\eta_{9}+\eta_{5}\eta_{7}\eta_{8}-\eta_{6}\eta_{7}^{2},$$

$$Q_{5}=t_{1}\eta_{4}^{2}+(t_{2}\eta_{5}+2t_{3}\eta_{6})\eta_{4}+t_{4}\eta_{5}^{2}+2t_{5}\eta_{5}\eta_{6}+t_{6}\eta_{6}^{2},$$

$$t_{1}=\eta_{9}^{2}-\eta_{2}\eta_{3},\;t_{2}=2\eta_{3}\eta_{7}-2\eta_{8}\eta_{9},\;t_{3}=\eta_{2}\eta_{8}-\eta_{7}\eta_{9},$$

$$t_{4}=\eta_{8}^{2}-\eta_{1}\eta_{3},\;t_{5}=\eta_{1}\eta_{9}-\eta_{7}\eta_{8},\;t_{6}=\eta_{1}\eta_{2}-\eta_{7}^{2}.$$

**Note 1:** We can generate many more estimators from a generalized class of estimators described in (43), are given below:

1. Put *λ*_1*c*_ = 0 and *λ*_2*c*_ = 0 in (43), we get,

$${\hat{\sigma }}_{I(P_{1})}^{2}=[\frac{K_{1c}}{H}\{s_{z}^{2}-\alpha^{2}\sigma_{S}^{2}H^{*}-{\bar{z}}^{2}H^{**}\}+K_{2c}(\sigma_{x_{1}}^{2}-s_{x_{1}}^{2})+K_{3c}(\sigma_{x_{2}}^{2}-s_{x_{2}}^{2})].$$

2. Put *λ*_1*c*_ = 1 and *λ*_2*c*_ = 1 in (43), we get,

$${\hat{\sigma }}_{I(P_{2})}^{2}=[\frac{K_{1c}}{H}\{s_{z}^{2}-\alpha^{2}\sigma_{S}^{2}H^{*}-{\bar{z}}^{2}H^{**}\}+K_{2c}(\sigma_{x_{1}}^{2}-s_{x_{1}}^{2})+K_{3c}(\sigma_{x_{2}}^{2}-s_{x_{2}}^{2})]$$

$$[\left\{\exp(\frac{\sigma_{x_{1}}^{2}-s_{x_{1}}^{2}}{\sigma_{x_{1}}^{2}+s_{x_{2}}^{2}})\}(\frac{\sigma_{x_{2}}^{2}}{s_{x_{2}}^{2}}\right)].$$

3. Put *λ*_1*c*_ = 1 and *λ*_2*c*_ = 0 in (43), we get,

$${\hat{\sigma }}_{I(P_{3})}^{2}=[\frac{K_{1c}}{H}\{s_{z}^{2}-\alpha^{2}\sigma_{S}^{2}H^{*}-{\bar{z}}^{2}H^{**}\}+K_{2c}(\sigma_{x_{1}}^{2}-s_{x_{1}}^{2})+K_{3c}(\sigma_{x_{2}}^{2}-s_{x_{2}}^{2})][\exp(\frac{\sigma_{x_{1}}^{2}-s_{x_{1}}^{2}}{\sigma_{x_{1}}^{2}+s_{x_{2}}^{2}})].$$

4. Put *λ*_1*c*_ = 0 and *λ*_2*c*_ = 1 in (43), we get,

$${\hat{\sigma }}_{I(P_{4})}^{2}=[\frac{K_{1c}}{H}\{s_{z}^{2}-\alpha^{2}\sigma_{S}^{2}H^{*}-{\bar{z}}^{2}H^{**}\}+K_{2c}(\sigma_{x_{1}}^{2}-s_{x_{1}}^{2})+K_{3c}(\sigma_{x_{2}}^{2}-s_{x_{2}}^{2})][\frac{\sigma_{x_{2}}^{2}}{s_{x_{2}}^{2}}].$$

5. Put *λ*_1*c*_ = −1 and *λ*_2*c*_ = −1 in (43), we get,

$${\hat{\sigma }}_{I(P_{5})}^{2}=[\frac{K_{1c}}{H}\{s_{z}^{2}-\alpha^{2}\sigma_{S}^{2}H^{*}-{\bar{z}}^{2}H^{**}\}+K_{2c}(\sigma_{x_{1}}^{2}-s_{x_{1}}^{2})+K_{3c}(\sigma_{x_{2}}^{2}-s_{x_{2}}^{2})]$$

$$[{\left\{\exp(\frac{\sigma_{x_{1}}^{2}-s_{x_{1}}^{2}}{\sigma_{x_{1}}^{2}+s_{x_{2}}^{2}})\right\}}^{-1}{\left(\frac{\sigma_{x_{2}}^{2}}{s_{x_{2}}^{2}}\right)}^{-1}].$$

6. Put *λ*_1*c*_ = −1 and *λ*_2*c*_ = 0 in (43), we get,

$${\hat{\sigma }}_{I(P_{6})}^{2}=[\frac{K_{1c}}{H}\{s_{z}^{2}-\alpha^{2}\sigma_{S}^{2}H^{*}-{\bar{z}}^{2}H^{**}\}+K_{2c}(\sigma_{x_{1}}^{2}-s_{x_{1}}^{2})+K_{3c}(\sigma_{x_{2}}^{2}-s_{x_{2}}^{2})]$$

$$[\exp{\left(\frac{\sigma_{x_{1}}^{2}-s_{x_{1}}^{2}}{\sigma_{x_{1}}^{2}+s_{x_{2}}^{2}}\right)}^{-1}].$$

7. Put *λ*_1*c*_ = 0 and *λ*_2*c*_ = −1 in (43), we get,

$${\hat{\sigma }}_{I(P_{7})}^{2}=[\frac{K_{1c}}{H}\{s_{z}^{2}-\alpha^{2}\sigma_{S}^{2}H^{*}-{\bar{z}}^{2}H^{**}]+K_{2c}(\sigma_{x_{1}}^{2}-s_{x_{1}}^{2})+K_{3c}(\sigma_{x_{2}}^{2}-s_{x_{2}}^{2})][{\left(\frac{\sigma_{x_{2}}^{2}}{s_{x_{2}}^{2}}\right)}^{-1}].$$

8. Put *λ*_1*c*_ = −1 and *λ*_2*c*_ = 1 in (43), we get,

$${\hat{\sigma }}_{I(P_{8})}^{2}=[\frac{K_{1c}}{H}\{s_{z}^{2}-\alpha^{2}\sigma_{S}^{2}H^{*}-{\bar{z}}^{2}H^{**}\}+K_{2c}(\sigma_{x_{1}}^{2}-s_{x_{1}}^{2})+K_{3c}(\sigma_{x_{2}}^{2}-s_{x_{2}}^{2})]$$

$$[{\left\{\exp(\frac{\sigma_{x_{1}}^{2}-s_{x_{1}}^{2}}{\sigma_{x_{1}}^{2}+s_{x_{2}}^{2}})\right\}}^{-1}(\frac{\sigma_{x_{2}}^{2}}{s_{x_{2}}^{2}})].$$

9. Put *λ*_1*c*_ = 1 and *λ*_2*c*_ = −1 in (43), we get,

$${\hat{\sigma }}_{I(P_{9})}^{2}=[\frac{K_{1c}}{H}\{s_{z}^{2}-\alpha^{2}\sigma_{S}^{2}H^{*}-{\bar{z}}^{2}H^{**}\}+K_{2c}(\sigma_{x_{1}}^{2}-s_{x_{1}}^{2})+K_{3c}(\sigma_{x_{2}}^{2}-s_{x_{2}}^{2})]$$

$$[\{\exp(\frac{\sigma_{x_{1}}^{2}-s_{x_{1}}^{2}}{\sigma_{x_{1}}^{2}+s_{x_{2}}^{2}})\}{\left(\frac{\sigma_{x_{2}}^{2}}{s_{x_{2}}^{2}}\right)}^{-1}].$$

**Note 2:** We can generate many more estimators from a generalized class of estimators given in

(62) are described below:

1. Put *π*_1_ = *π*_2_ = *π*_3_ = 1 in (62), we get,

$${\hat{\sigma }}_{P_{1}}^{2}=[\frac{L_{1}}{H}\{s_{z}^{2}-\alpha^{2}\sigma_{S}^{2}H^{*}-{\bar{z}}^{2}H^{**}\}\{\exp(\frac{\sigma_{x_{1}}^{2}-s_{x_{1}}^{2}}{\sigma_{x_{1}}^{2}+s_{x_{1}}^{2}})\}]$$

$$+L_{2}\{\exp(\frac{s_{x_{1}}^{2}-\sigma_{x_{1}}^{2}}{s_{x_{1}}^{2}+\sigma_{x_{1}}^{2}})\}+L_{3}\{\exp(\frac{s_{x_{2}}^{2}-\sigma_{x_{2}}^{2}}{s_{x_{2}}^{2}+\sigma_{x_{2}}^{2}})\}$$

2. Put *π*_1_ = 0 and *π*_2_ = *π*_3_ = 1 in (62), we get,

${\hat{\sigma }}_{P_{2}}^{2}=[\frac{L_{1}}{H}\{s_{z}^{2}-\alpha^{2}\sigma_{S}^{2}H^{*}-{\bar{z}}^{2}H^{**}\}]+L_{2}\{\exp(\frac{s_{x_{1}}^{2}-\sigma_{x_{1}}^{2}}{s_{x_{1}}^{2}+\sigma_{x_{1}}^{2}})\}+L_{3}\{\exp(\frac{s_{x_{2}}^{2}-\sigma_{x_{2}}^{2}}{s_{x_{2}}^{2}+\sigma_{x_{2}}^{2}})\}$

3. Put *π*_1_ = −1 and *π*_2_ = *π*_3_ = 1 in (62), we get, ${\hat{\sigma }}_{P_{3}}^{2}=[\frac{L_{1}}{H}\{s_{z}^{2}-\alpha^{2}\sigma_{S}^{2}H^{*}-{\bar{z}}^{2}H^{**}\}{\left\{\exp(\frac{\sigma_{x_{1}}^{2}-s_{x_{1}}^{2}}{\sigma_{x_{1}}^{2}+s_{x_{1}}^{2}})\right\}}^{-1}]$ $+L_{2}\{\exp(\frac{s_{x_{1}}^{2}-\sigma_{x_{1}}^{2}}{s_{x_{1}}^{2}+\sigma_{x_{1}}^{2}})\}+L_{3}\{\exp(\frac{s_{x_{2}}^{2}-\sigma_{x_{2}}^{2}}{s_{x_{2}}^{2}+\sigma_{x_{2}}^{2}})\}$

4. Put *π*_1_ = 0.5 and *π*_2_ = *π*_3_ = 1 in (62), we get, ${\hat{\sigma }}_{P_{4}}^{2}=[\frac{L_{1}}{H}\{s_{z}^{2}-\alpha^{2}\sigma_{S}^{2}H^{*}-{\bar{z}}^{2}H^{**}\}{\left\{\exp(\frac{\sigma_{x_{1}}^{2}-s_{x_{1}}^{2}}{\sigma_{x_{1}}^{2}+s_{x_{1}}^{2}})\right\}}^{0.5}]$ $+L_{2}\{\exp(\frac{s_{x_{1}}^{2}-\sigma_{x_{1}}^{2}}{s_{x_{1}}^{2}+\sigma_{x_{1}}^{2}})\}+L_{3}\{\exp(\frac{s_{x_{2}}^{2}-\sigma_{x_{2}}^{2}}{s_{x_{2}}^{2}+\sigma_{x_{2}}^{2}})\}$

5. Put *π*_1_ = 0 and *π*_2_ = *π*_3_ = 1 in (62), we get, ${\hat{\sigma }}_{P_{5}}^{2}=[\frac{L_{1}}{H}\{s_{z}^{2}-\alpha^{2}\sigma_{S}^{2}H^{*}-{\bar{z}}^{2}H^{**}\}]+L_{2}{\left\{\exp(\frac{s_{x_{1}}^{2}-\sigma_{x_{1}}^{2}}{s_{x_{1}}^{2}+\sigma_{x_{1}}^{2}})\right\}}^{-1}+L_{3}{\left\{\exp(\frac{s_{x_{2}}^{2}-\sigma_{x_{2}}^{2}}{s_{x_{2}}^{2}+\sigma_{x_{2}}^{2}})\right\}}^{-1}$

Put *π*_1_ = 0, *π*_2_ = 1 and *π*_3_ = −1 in (62), we get, ${\hat{\sigma }}_{P_{6}}^{2}=[\frac{L_{1}}{H}\{s_{z}^{2}-\alpha^{2}\sigma_{S}^{2}H^{*}-{\bar{z}}^{2}H^{**}\}]+L_{2}\{\exp(\frac{s_{x_{1}}^{2}-\sigma_{x_{1}}^{2}}{s_{x_{1}}^{2}+\sigma_{x_{1}}^{2}})\}+L_{3}{\left\{\exp(\frac{s_{x_{2}}^{2}-\sigma_{x_{2}}^{2}}{s_{x_{2}}^{2}+\sigma_{x_{2}}^{2}})\right\}}^{-1}$

6. Put *π*_1_ = 0, *π*_2_ = −1 and *π*_3_ = 1 in (62), we get, ${\hat{\sigma }}_{P_{7}}^{2}=[\frac{L_{1}}{G}\{s_{z}^{2}-\alpha^{2}\sigma_{S}^{2}H^{*}-{\bar{z}}^{2}H^{**}\}]+L_{2}{\left\{\exp(\frac{s_{x_{1}}^{2}-\sigma_{x_{1}}^{2}}{s_{x_{1}}^{2}+\sigma_{x_{1}}^{2}})\right\}}^{-1}+L_{3}\{\exp(\frac{s_{x_{2}}^{2}-\sigma_{x_{2}}^{2}}{s_{x_{2}}^{2}+\sigma_{x_{2}}^{2}})\}$

## 7. Simulation study

In this section, a simulation study is conducted to validate the efficiency of estimators. We consider three populations of size *N* = 500 each from three bivariate normal populations having mean and covariance matrices, are given below:

**Population-I**

$$\mu =[\begin{array}{c} 5\\ 5\\ 5 \end{array}],\;\mathrm{and}\;\sum =[\begin{array}{ccc} 10 & 3 & 2.9\\ 3 & 2 & 1.1\\ 2.9 & 1.1 & 2 \end{array}]$$

*μ*_*y*_ = 5.10938, $\mu_{x_{1}}=5.030367,$ $\mu_{x_{2}}=5.058825,$ $\sigma_{y}^{2}=9.462803,$ $\sigma_{S}^{2}=0.2636628,$ $\sigma_{T}^{2}=0.2198689,$ $\sigma_{yx_{1}}=2.669449,$ $\sigma_{yx_{2}}=2.897638,$ $\sigma_{x_{1}x_{2}}=1.111704,$ $\rho_{yx_{1}}=0.63388,$ $\rho_{yx_{2}}=0.65747,$ $\rho_{x_{1}x_{2}}=0.56680.$

**Population-II**

$$\mu =[\begin{array}{c} 5\\ 5\\ 5 \end{array}],\;\mathrm{and}\;\sum =[\begin{array}{ccc} 6 & 3 & 2.9\\ 3 & 2 & 1.1\\ 2.9 & 1.1 & 2 \end{array}]$$

*μ*_*y*_ = 5.084729, $\mu_{x_{1}}=5.04074,$ $\mu_{x_{2}}=5.057682,$ $\sigma_{y}^{2}=5.677682,$ $\sigma_{S}^{2}=0.2636628,$ $\sigma_{T}^{2}=0.2198689,$ $\sigma_{yx_{1}}=2.750379,$ $\sigma_{yx_{2}}=2.888632,$ $\sigma_{x_{1}x_{2}}=1.065366,$ $\rho_{yx_{1}}=0.8504945,$ $\rho_{yx_{2}}=0.8499048,$ $\rho_{x_{1}x_{2}}=0.5503346.$

**Population-III**

$$\mu =[\begin{array}{c} 5\\ 5\\ 5 \end{array}],\;\mathrm{and}\;\sum =[\begin{array}{ccc} 9 & 1.8 & 1.5\\ 1.8 & 4 & 0.8\\ 1.5 & 0.8 & 4 \end{array}]$$

*μ*_*y*_ = 5.103771, $\mu_{x_{1}}=5.0163,$ $\mu_{x_{2}}=5.066954,$ $\sigma_{y}^{2}=8.516522,$ $\sigma_{S}^{2}=0.2636628,$ $\sigma_{T}^{2}=0.2198689,$ $\sigma_{yx_{1}}=1.410978,$ $\sigma_{yx_{2}}=1.705392,$ $\sigma_{x_{1}x_{2}}=0.9903949,$ $\rho_{yx_{1}}=0.2434704,$ $\rho_{yx_{2}}=0.2892275,$ $\rho_{x_{1}x_{2}}=0.2468379.$ Covariance matrices show the distribution of the sensitive variable *Y* and the auxiliary variables *X*_1_ and *X*_2_. The response variable is *Z* = *TY*+*S*, where the scrambling variables *S* and *T* are distributed normally with mean 0 and variance 1 respectively. *MSE* values of different estimators given in Tables 1–4 are based on the various models as discussed earlier.

**Table 1:** *MSE* values of Gupta et. al [10] estimator under Diana and Perri [8] model.

Estimator	Variable	Population I	Population II	Population III
${\hat{\sigma }}_{G(0)}^{2}$	-	11.8287	5.6344	9.6159
${\hat{\sigma }}_{G(R)C}^{2}$	*X*_1_	12.8786	4.8581	12.4518
${\hat{\sigma }}_{G(G)C}^{2}$	*X*_1_	10. 8234	4.7481	9.6087
${\hat{\sigma }}_{D_{1}}^{2}$	*X*_1_	10.6410	4.5889	9.3315
${\hat{\sigma }}_{D_{2}}^{2}$	*X*_1_,*X*_2_	9.9920	4.1065	8.6678

**Table 2:** *MSE* values of Saleem et al. [20] estimator under generalized model using two auxiliary variables.

Estimator	Constant (*λ*_1*c*_,*λ*_2*c*_)	Population I	Population II	Population III
${\hat{\sigma }}_{I(P_{1})}^{2}$	(0,0)	9.6993	4.1016	8.6678
${\hat{\sigma }}_{I(P_{2})}^{2}$	(1,1)	9.5721	3.9831	8.4982
${\hat{\sigma }}_{I(P_{3})}^{2}$	(1,0)	9. 5785	4.0303	8.5714
${\hat{\sigma }}_{I(P_{4})}^{2}$	(0,1)	9.6147	4.0362	8.5781
${\hat{\sigma }}_{I(P_{5})}^{2}$	(−1,−1)	10.3291	4.3284	9.2236
${\hat{\sigma }}_{I(P_{6})}^{2}$	(−1,0)	9.9328	4.2015	8.8528
${\hat{\sigma }}_{I(P_{7})}^{2}$	(0,−1)	10.1375	4.2530	9.0584
${\hat{\sigma }}_{I(P_{8})}^{2}$	(−1,1)	9.7846	4.1226	8.7519
${\hat{\sigma }}_{I(P_{9})}^{2}$	(1,−1)	9.9854	4.1796	8.9924

**Table 3:** *MSE* values of Azeem et al. [6] estimator under a given model.

Estimator	Constant	Population I	Population II	Population III
${\hat{\sigma }}_{y(A)C}^{2}$	*l* = 0	12.091	6.242	10.420
${\hat{\sigma }}_{R(A)C}^{2}$	*l* = 0	29.576	21.993	26.908
${\hat{\sigma }}_{y(A)C}^{2}$	*l* = 1	6.619	2.312	5.329
${\hat{\sigma }}_{R(A)C}^{2}$	*l* = 1	13.742	9.264	12.034

**Table 4:** *MSE* values of proposed estimator under generalized model using two auxiliary variables.

Estimator	Constant (*π*_1_,*π*_2_,*π*_3_)	Population I	Population II	Population III
${\hat{\sigma }}_{P_{1}}^{2}$	(0,0,0)	0.59781	0.23416	0.35849
${\hat{\sigma }}_{P_{2}}^{2}$	(0,1,1)	0.63575	0.22071	0.40495
${\hat{\sigma }}_{P_{3}}^{2}$	(−1,1,1)	0.60196	0.19113	0.42406
${\hat{\sigma }}_{P_{4}}^{2}$	(0.5,1,1)	0.62529	0.23010	0.38420
${\hat{\sigma }}_{P_{5}}^{2}$	(0,−1,−1)	0.48514	0.14678	0.37914
${\hat{\sigma }}_{P_{6}}^{2}$	(0,1,−1)	0.30909	0.12798	0.34077
${\hat{\sigma }}_{P_{7}}^{2}$	(0,−1,1)	0.30950	0.12129	0.32906

In Table 1, we observed that the difference estimator ${\hat{\sigma }}_{D_{2}}^{2}$ has the least variance as compared to other considered estimators under all Populations I, II and III. Table 2 gives the *MSE* values of Saleem et al. [20] model for different values of *λ*_*i*_(*i* = 1,2) which indicates that Saleem et al. [20] estimators are generally perform better as compared to the estimators given in Table 1. Table 3 provides the *MSE* values of correct version of Azeem et al. [6] model. Table 4 provides the least *MSE* values of the proposed estimator under different values of *π*_*i*_(*i* = 1,2,3) as compared to all other considered estimators given in Tables 1–3. The ratio estimator ${\hat{\sigma }}_{R(A)C}^{2}$ for *l* = 0 shows poor performance in Table 3 due to a model suggested by Azeem et al. [6]. The proposed estimator is based on the corrected model which earlier used by Saleem et al. [20] and play a major role in reduction of the *MSE*s under different situations.

## 8. Conclusion

Using the generalized RRM, we proposed an enhanced general class of difference-in-exponential ratio-product type estimators in estimating the finite population variance utilizing two auxiliary variables. We adjusted the bias and *MSE* expressions of the Gupta et al. [10], Saleem et al. [20] and Azeem et al. [6] estimators. The usefulness of the proposed estimator is demonstrated by a simulated study that uses the RRM in estimating the finite population variance using three data sets. The performance of the proposed estimator (see Table 4) is superior under different scenarios as compared to all other considered estimators (see Tables 1–3). The reduction in *MSE* is due to the right choice of RRM, so proposed estimator may be useable in all sorts of real data sets because of flexibility in accommodating both positive and negative correlation coefficients. The key findings include: (i) Many new estimators can be generated from a proposed generalized class of estimators. (ii) The proposed class of estimators is restricted to two auxiliary variables but by employing other suitable designs, it may be expanded to include multi-auxiliary variables. (iii) Use of appropriate RRMs, the efficiency of the suggested class of estimators can be improved.
